# A Systematic Review of Childhood Diabetes Research in the Middle East Region

**DOI:** 10.3389/fendo.2019.00805

**Published:** 2019-11-19

**Authors:** Saras Saraswathi, Sara Al-Khawaga, Naser Elkum, Khalid Hussain

**Affiliations:** ^1^Division of Endocrinology, Department of Pediatrics, Sidra Medicine, Doha, Qatar; ^2^College of Health & Life Sciences, Hamad Bin Khalifa University, Qatar Foundation, Education City, Doha, Qatar; ^3^Biostatistics Section, Clinical Research Center, Research Services, Sidra Medicine, Doha, Qatar

**Keywords:** T1DM, Middle-East, childhood, MODY, insulin-resistance, prevention, epidemiology, registry

## Abstract

**Background:** Diabetes mellitus (DM) is a common chronic disorder in children and is caused by absolute or relative insulin deficiency, with or without insulin resistance. There are several different forms of childhood DM. Children can suffer from neonatal diabetes mellitus (NDM), type 1 diabetes (T1DM), type 2 diabetes (T2DM), Maturity Onset Diabetes of the Young (MODY), autoimmune monogenic, mitochondrial, syndromic and as yet unclassified forms of DM. The Middle East has one of the highest incidences of several types of DM in children; however, it is unclear whether pediatric diabetes is an active area of research in the Middle East and if ongoing, which research areas are of priority for DM in children.

**Objectives:** To review the literature on childhood DM related to research in the Middle East, summarize results, identify opportunities for research and make observations and recommendations for collaborative studies in pediatric DM.

**Methods:** We conducted a thorough and systematic literature review by adhering to a list recommended by PRISMA. We retrieved original papers written in English that focus on childhood DM research, using electronic bibliographic databases containing publications from the year 2000 until October 2018. For our final assessment, we retrieved 429 full-text articles and selected 95 articles, based on our inclusion and exclusion criteria.

**Results:** Our literature review suggests that childhood DM research undertaken in the Middle East has focused mainly on reporting retrospective review of case notes, a few prospective case studies, systemic reviews, questionnaire-based studies, and case reports. These reported studies have focused mostly on the incidence/prevalence of different types of DM in childhood. No studies report on the establishment of National Childhood Diabetes Registries. There is a lack of consolidated studies focusing on national epidemiology data of different types of childhood DM (such as NDM, T1DM, T2DM, MODY, and syndromic forms) and no studies reporting on clinical trials in children with DM.

**Conclusions:** Investing in and funding basic and translational childhood diabetes research and encouraging collaborative studies, will bring enormous benefits financially, economically, and socially for the whole of the Middle East region.

## Introduction: Overview of Childhood Diabetes Mellitus

### Burden of Diabetes

Recent trends have indicated that the incidence of diabetes is increasing rapidly worldwide (1), with a dramatic increase in prevalence in the Middle Eastern countries, among adults (2) and in children alike (3, 4). This trend is evidenced and emphasized by a 3% increase in the occurrence of this disease in children, in whom it manifests itself in many debilitating ways (1, 4).

According to the IDF Diabetes Atlas—Seventh Edition (3), the number of children (0–14 years) with T1DM in the Middle East and North Africa (MENA) Region is 60,700 and the number of newly diagnosed children each year is 10,200. According to the IDF Diabetes Atlas—eighth Edition (4), the number of adolescents in Qatar with T1DM is 592, and the number of newly diagnosed children and adolescents, per 100,000 children per year is 12.2. Other data, such as for undiagnosed cases of DM, mortality rates, and health care expenditure due to DM are given only for adults. The total health expenditure for the adult population is expected to go from 17.1 billion in 2015 to 30 billion in 2040 (3). There are many consequences due to DM and complications such as stroke, blindness, heart attacks, kidney failure, and amputations can occur. There is a major social cost due to this disease and the risks increase with age, genetic factors, and family history. Those with diabetes are likely to have double the amount of health expenditure than others (3).

It is therefore important to identify the causes of this trend and develop newer therapies through improved research that could result in the development of better treatment and care. Hence in this study, we thoroughly reviewed the published literature to try and understand the types of research reported in childhood DM in the Middle East.

### Nature of the Disease—Diabetes

Diabetes mellitus (DM) is a complex, chronic metabolic condition that results in hyperglycemia and is caused by an absolute or relative insulin deficiency with or without insulin resistance. Neonatal diabetes mellitus (NDM) occurs before 6 months of age and is relatively rare. Although Type 1 DM (T1DM) is the commonest form of DM observed in children, Type 2 DM (T2DM) is becoming more prevalent for this age group where the rising numbers are mostly driven by the obesity epidemic (5). Maturity Onset Diabetes of the Young (MODY) can also present during childhood. Autoimmune monogenic forms of DM are a relatively new group of diseases described in children associated with multiple autoimmunities. Other rare forms of DM observed during childhood include mitochondrial DM, syndromic forms of DM and as yet unclassified forms. Cystic Fibrosis (CF) related to diabetes, also known as (CFRD) develops in many patients over time (6). Considering that current epidemiology data about CF in the Middle East is between one in 2000 and 5,800 live births, this is an important area of research (7).

#### Type 1 Diabetes Mellitus

In a six-center study conducted in the USA, 80% of DM cases were for T1DM for those <9 years of age and 6–76% for those between 10 and 19 years of age (5). T1DM accounts for nearly two-thirds of newly diagnosed patients in the United States, who are <19 years of age (5). In 2006, the number of children with T1DM was estimated by the International Federation of Diabetes (IDF) to be 440,000, an annual increase of 3%, with 70,000 newly diagnosed cases per year (4). Furthermore, the prevalence of cases in individuals younger than 15 years of age is estimated to rise by 70% (8, 9). These epidemiological data suggest an “accelerating” epidemic and serves as a useful indicator of the future burden of T1DM.

T1DM is the most common form of childhood DM and is due to a combinations of factors, such as defective autoimmunity, genetics, and environmental factors. T1DM occurs during early through mid-childhood when pancreatic beta-cells are destroyed, as a result of an autoimmune process, resulting in a lack of insulin production. The autoantibodies facilitate the destruction of the beta-cells over the years, which results in metabolic abnormalities ranging from asymptomatic hyperglycemia to frank DM. The underlying genetic or other mechanisms that trigger the onset of T1DM are not known, but ~50% of the familial clustering of genes, which increase the susceptibility risk of inheriting T1DM, are located within or in the human leukocyte antigen (HLA) complex on chromosome 6 (10). The highest risk haplotypes (such as HLA-DR4-DQ8 and DR3-DQ2) are known to confer the greatest risk for developing T1DM, particularly when occurring together. However, ~10% of patients with DM do not carry any of these high-risk HLA class II haplotypes (10).

Autoimmunity in T1DM relies on the detection of insulitis, islet cell antibodies (ICA) and activated beta-cell-specific T lymphocytes (11). These beta-cell-specific autoantibodies are thought to be the molecular markers of the diabetogenic process. Although the type of antibody a patient has is an important indicator of the disease, a patient's progression to develop DM can be predicted more accurately if they have an increasing number of antibodies (12). Insulin Autoantibodies (IAA) tend to appear early on in a child's life with other antibodies [such as Glutamic Acid Decarboxylase (GAD65), insulinoma-2 antigen antibodies (IA-2A), and Zinc transporter-8 (ZnT8)] appearing later (13). The presence of one or more of these autoantibodies increases the risk of developing T1DM (12).

A recent study (14) estimated the prevalence ZnT8A in juvenile-onset T1DM, to establish its utility as a standalone marker for T1DM in subjects who tested negative for other antibodies. This study (14) also investigated ZnT8A's co-existence with other antibodies such as GADA and insulinoma-2 antigen antibodies (IA-2A). When compared to other antibodies, prevalence of ZnT8a (31.8%) was lower than that of GADA (64.7%), but higher than IA2A (19.3%) (14). While 45% of newly diagnosed patients tested positive for ZnT8A, it was uniquely present in 26% of these patients (where patients tested negative for GADA and IA-2A) (14), which was a much higher value when compared to the unique presense of IA-2A in just two patients. Hence, this study (14) found that the combined presence of GADA and ZnT8A were better predictors of T1DM (at 97%) when compared to IA-2A. In one study (15) 32% of cases (in 12 out of 38 children in the study) with T2DM were antibody positive, where the patients were primarily obese and females of pubertal age.

#### Type 2 Diabetes Mellitus

T2DM is a chronic disease, which is complex and heterogeneous in its manifestations (16). Its risk factors vary with environmental, social and behavioral patterns and are also susceptible to genetic variations. Childhood obesity is the primary cause of T2DM at a young age. The increased prevalence of obesity over the last two decades has increased the number of patients who have T2DM. In the Arab world, it is estimated that the number of diabetic patients (adults and children) will increase by 96.2% by 2035, mostly driven by the increase in T2DM (17). Although genetic factors may be contributing for the increased number of T2DM cases being diagnosed in children in the Middle East, changing the lifestyle that has resulted in urbanization, unhealthy and sedentary life and obesity due to rich food intake, have also contributed to the increased prevalence of T2DM (18).

#### Maturity Onset Diabetes of the Young (MODY)

Maturity Onset Diabetes of the Young (MODY) occurs due to defects in a single gene. It can affect about 4% of diabetes patients. MODY generally occurs before the age of 25 and typically several family members might be affected (autosomal dominant inheritance pattern). Mutations in 12 different genes have been identified as causative of MODY (19). Encoding the commonest causes of MODY are mutations in the genes Hepatic Nuclear Factor 1 Alpha (HNF1A) and HNF4A and the enzyme Glucokinase (GCK) (15, 19, 20). MODY is commonly misdiagnosed as T1DM or T2DM. A diagnosis of MODY based on genetic testing can benefit patients as some of these patients can be managed by oral sulphonylreas (21).

#### Neonatal Diabetes

Neonatal diabetes mellitus (NDM) is classified as an early-onset (below 6 months of life) and rare form of diabetes that affects newborns with an increased rate of incidence of 1:90,000 which is nearly four times the number previously reported (19, 22, 23). Transient NDM (TNDM) and permanent NDM (PNDM) are the two main forms of NDM, which are classified according to the duration of the insulin dependency. About 50–60% of the cases are TNDM and the disease is generally expected to resolve in <18 months (24).

NDM in western countries is caused by defects in the *KCNJ11/ABCC8* genes, which encode for the pancreatic beta-cell K_ATP_ channel (25). However, NDM in the Middle East, among Arabic populations has a different genetic basis when compared to westerners (26). Mutations in the Glucokinase (*GCK*) gene is a frequent form of NDM in countries with high consanguinity rate since a homozygous or a compound heterozygous mutation in this gene leads to complete glucokinase deficiency that results in PNDM (27). Higher rate of consanguinity among Arabs makes PNDM mostly likely to occur as part of a recessively inherited syndrome such as Wolcott-Rallison syndrome, Fanconi-Bickel syndrome, and thiamine-responsive megaloblastic anemia and hearing loss (also known as Rogers's syndrome) (26).

#### Maternally Inherited Diabetes

Organelles such as the Mitochondria, contain circular DNA, called mtDNA. They are inherited through the maternal allele since they are present only in the oocytes. Defects in mtDNA are suspected to cause many diseases that include diabetes (28). The defective mtDNA can gradually cause damage to the beta-cells. The m.3243A>G mutation in the mtDNA (that codes for tRNA leucine) is the cause of this disease in over 85% of the patients. Since this disease is inherited only from the mother, it is called maternally inherited diabetes (29).

#### Syndromic Forms of Diabetes Mellitus

DM may also be associated with some rare syndromes involving other pancreatic features. Some of these rare syndromes include Wolfram (or DIDMOAD for diabetes insipidus, diabetes mellitus, optic atrophy, and deafness), Wolcott-Rallison, Alstrom, Bardet–Biedl, and Rogers's syndrome. Wolfram syndrome is the association between DM, diabetes insipidus, optic atrophy and sensorineural deafness (30), caused by defects in the WFS1 gene that is the negative regulator of endoplasmic reticulum signaling. Wolcott-Rallison occurs due to an autosomal recessive condition (that is rare), which results in an early presentation of DM accompanied by skeletal dysplasia, growth retardation, and multisystem clinical manifestations due to defects in the EIF2AK3 gene (31). Alstrom syndrome results in loss of vision and hearing, dilated cardiomyopathy and DM (32), caused by defective ALMS1 gene. Rogers's syndrome is due to defects in the SLC19A2 gene. Rogers's syndrome comprises of megaloblastic anemia, DM and sensorineural deafness (33). The clinical features of Bardet-Biedl include rod-cone dystrophy, with childhood-onset visual loss preceded by night blindness, postaxial polydactyly, truncal obesity, and DM (34).

#### Autoimmune Monogenic DM

Autoimmune monogenic DM is a relatively new group of diseases, where DM is associated with multiple autoimmune defects in these four genes: autoimmune regulator (AIRE) part of autoimmune polyendocrine syndromes (APS) (35, 36), forkhead box P3 (FOXP3) (37), sirtuin 1 (SIRT1) (38), and signal transducer and activator of transcription 3 (STAT3) (39). Defects that occur in any one of these genes can cause autoimmune diabetes that can affect many other organs, suggesting that in some patients, diabetes may be part of a complex autoimmune process involving multiple organs.

### Rationale and Scope of This Study

There are several reviews published in the literature which were specially tailored to look at studies published under DM in the Middle East (ME). Appendix A lists nine of these reviews. The first review investigates the burden imposed by DM on the Saudi population and recommends ways to mitigate this disease (17). Other reviews discuss the increasing prevalence of DM and advocate a better understanding of the epidemiology and early detection and control of DM among subgroups in the population (40–42). A third review (43) recognizes the paucity of DM related research and publications in the Middle East when compared to other advanced countries. The recommendations of this review are also along the lines of control of DM through diet and changes in lifestyle (43). A fourth review (44) recognizes that consanguineous marriages, a customary practice peculiar to the Arabic regions, can predispose the population to novel and unique genetic mutations that can cause DM. They emphasize the need for establishing a diabetes registry (44), based on Arab populations that encompasses 22 Arabic speaking countries. They reiterate that the information related to genetic variants in non-Arabic populations discovered elsewhere, will probably be irrelevant for understanding the epidemiology and underlying genetics in the ME populations. They point out that very few registries are currently available among the ME countries and is one of the studies that advocate a collaborative approach to research in DM. The remaining two reviews report on the alarming trends in DM which seem to affect an increasing number of urban, female and younger populations (45, 46).

Although many review papers have been published, they are limited to certain types of diabetes such as T1DM, T2DM (40, 47), and diabetic ketoacidosis (DKA) (48). There is no mention of other types of diabetes such as MODY, autoimmune monogenic forms and other rare forms such as mitochondrial DM and syndromic forms that can affect children. Some of these reviews are limited to only certain ME countries, where the studies took place (43, 49). The nature of these articles are quite diverse but are mostly limited to observational studies that advocate disease control. Some articles provide information on DM in areas outside of the Middle East (50, 51) and some of them do not cover pediatric populations exclusively (41–45). Hence there is a need for a comprehensive review that encompasses all the topics of interest discussed above that pertain only to children and adolescents.

Our study aims to cover all manifestations of childhood diabetes research that has been reported in the Middle East countries. It will investigate the state of research for all the sub-types of DM disease in children, to provide a consolidated and comprehensive view of the current state of affairs in DM. We are not aware of any previously published systematic reviews that have addressed these fundamental research questions on childhood DM in the Middle East.

### Aims of This Study

Although childhood DM is common in the Middle East, T1DM, NDM, and syndromic forms of diabetes have a high incidence rate in this region (17, 45). The prevalence of diabetes has steeply increased over the years in the Middle East and the region has been increasingly burdened with childhood DM. The existing reviews do not include studies on all types of DM and there is very little information on studies that investigate the molecular basis of the disease. Hence, we undertook a systematic review of publications that relate to research on childhood diabetes in the Middle East. The key questions we wanted to address were:

- What types (basic, clinical, and translational) of research has been reported in childhood DM?
- What impact does this research have on the local population of children in the Middle East?
- What research strategies are in place to tackle the burden of childhood DM in the Middle East?
- What funding opportunities are available for childhood DM research in the Middle East?
- What collaborations exist between different Middle Eastern countries in childhood DM research?

We hope to make recommendations and suggestions for collaborative research related to childhood DM in the Middle East, based on the knowledge gained from this study.

## Objectives

- To systematically review the literature on childhood (aged between 0 and 18 years) DM research in the Middle East region, published between the years 2000 and 2018.
- To summarize the results of studies reporting on childhood DM research in the Middle East.
- To identify key areas and opportunities for research in childhood DM in the Middle East.
- To make recommendations for collaborative research opportunities in childhood DM based on our identification of key areas that need attention to improve diabetic care.

## Methods

We aim to review the state of research in pediatric diabetes in the Middle East region. We broadly follow the guidelines provided by *Agency for Healthcare Research and Quality (AHRQ) Methods Guide* for this comparative effectiveness review (52, 53) and *Preferred Reporting Items for Systematic Reviews and Meta-Analyses (PRISMA)* (54).

### Literature Search Strategy and Study Selection

Initially, we identified our objectives (section Objectives) and predefined our search criteria for articles based on these objectives. Four months were earmarked for the literature search and collation of articles by two analysts (SS and SAK). Three months were earmarked for the analysis and review of the articles by senior authors (KH and NE).

We undertook an extensive literature search as suggested by PRISMA (54), to recover articles of primary interest that were published in English. We used the internet to search the electronic bibliographic databases for publications reporting research studies in the Middle East that addressed problems related to diabetes in children and adolescents. The dates included in the search for these studies were over a period of 18 years, between January 1st, 2000 and October 31st, 2018. Search terms and these search strategies are detailed in Table 1. Articles that had any of these search terms in their titles, abstracts or keywords list, were collected. EndNote©, a reference management software, was used to share and keep track of the titles and abstracts of articles of interest. A systematic list of articles detailing the eligibility/selection criteria for each of the articles was also maintained in Microsoft© Excel and categorized according to year of publication, age, study type, study design, study size, and prevalence of each subcategory of the disease.

**Table 1:** Search terms and search strategy.

**Search terms and search strategy**		
Publications	Medical databases such as PubMed and Medline of the National Institute of Health (NIH), Pubtator (a web-based tool that uses advanced text mining methods on PubMed), journals such as NEJM, BMJ, Web of Science, Embase **(**a biomedical literature database**)**, Science Direct, journals related to endocrinology, diabetes and metabolism, Google scholar, global and local pediatric publications in GCC (Gulf Cooperation Council)	
Population	“Children” OR “Adolescents” OR “Childhood” OR “Infants” OR “Teens” OR “Teenagers” OR “Youths”	
and	
Arab countries	“Qatar” OR “Saudi Arabia” OR “KSA” OR “Bahrain” OR “Emirate” OR “UAE” OR “Kuwait” OR “Oman” OR “Egypt” OR “Yemen” OR “Iran” OR “Iraq” OR “Arabian Gulf” OR “GCC” OR “Middle East” OR “Arab”	
and	
Outcome	Adolescent diabetes mellitus	Insulin-dependent diabetes
	Autoantibody and/or antibody	Ketoacidosis
	Autoimmune	MODY
	Childhood	Monogenic
	Childhood diabetes in developing countries	NDM
	Continuous glucose monitoring	Neonatal diabetes
	Diabetes mellitus	Non-insulin-dependent
	Diabetic complications	Prevalence
	Diabetic risk factors	Risk factors
	Diabetic syndrome	Risk of diabetes
	Endocrine and/or polyendocrine	Risk of diabetes in Arabian populations
	Epidemiology	Syndromic
	Gene mutations and/or mutations	T1D
	HLA and/or HLA haplotype	T1DM
	Hypoglycemia	T2DM
	IDDM and/or insulin-dependent diabetes mellitus	Type 1
	Incidence	Type 2
	Insulin pumps	Type 1 diabetes
	Insulin-dependent	Type 2 diabetes

**Figure 1:**
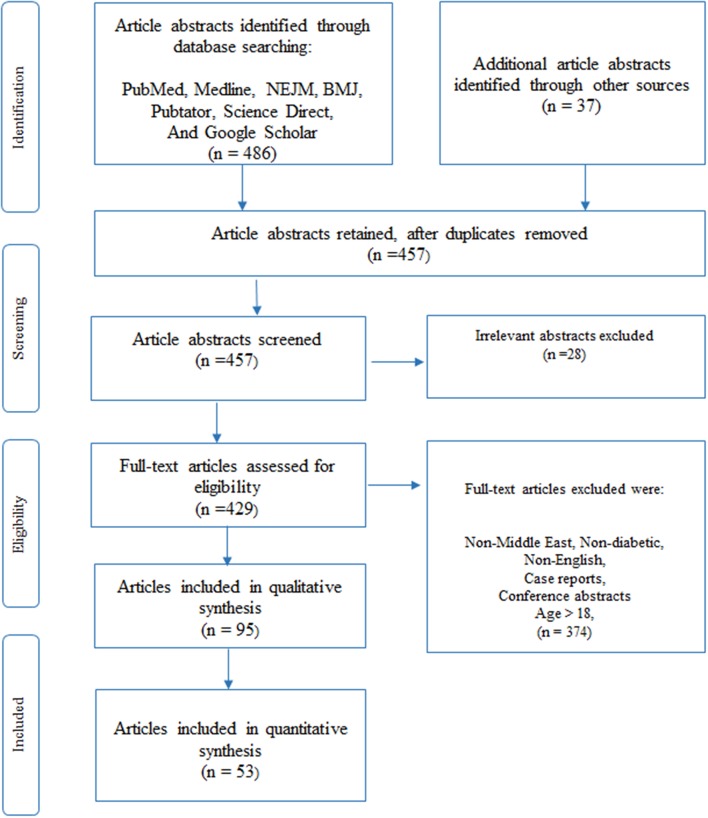
Article selection using PRISMA (54) methodology, as described under the Methods section, was used to select 95 articles for our review of which 53 articles relate to previous reviews (10), clinical and molecular research studies (43) in DM. The given figure is a modified version of the PRISMA methodology (54).

Four hundred and eighty-six (486) articles were initially identified through a database search in PubMed, Medline, NEJM, BMJ, Pubtator, Science Direct, and Google scholar (Figure 1). We obtained an additional thirty-seven (37) articles through other means such as Google search. Two analysts performed independent analysis of the titles and abstracts to eliminate articles that were unrelated or duplicated, to finally obtain four hundred and fifty-seven (457) abstracts. After removing another twenty-eight (28) irrelevant abstracts, we assessed four hundred and twenty-nine (429) full-text articles for inclusion criteria in this study.

Full-content articles were downloaded for the 429 abstracts and were examined and critically analyzed by two senior reviewers (KH and NE), to evaluate their suitability and to determine any bias in the selection of articles. Articles were removed if their content related to non-Middle-East regions, non-diabetic studies, written in a language other than in English, patient age was above 18, case reports or not peer-reviewed (conference presentations). Once agreement on the included articles was reached, data was extracted from the full-text articles. We used the PRISMA methodology (54) to select the final set of articles for qualitative synthesis in our review. Ninety-five (95) articles that included previously published reviews and reference materials were collected for qualitative synthesis, where the reviews were used only for content comparison. Finally, fifty-three (53) articles, that were published in the Middle East Region that pertained only to diabetic-related clinical or molecular studies were selected for quantitative synthesis (listed in Appendix B). Of these articles, nine (9) studies were previously published reviews that were used to determine their contribution to existing knowledge and to identify gaps that are to be filled. The remaining forty-four (44) articles were then separated into sub-groups according to the nature of the disease, as seen in subtitles in section Results of this paper.

### Inclusion Criteria

We included all articles that were published in the Arab countries that were related to diabetic studies on children below 18 years of age. We included only full-text publications and original articles that were published in the peer-reviewed journals (Table 1) between the years of 2000 and 2018. Articles that were printed locally in Arab countries (Table 1), in journals that related to diabetic studies on children below 18 years of age, were also included in our studies. The initial research included all types of research study designs such as meta-analysis, randomized controlled trials (RCT), observational studies, case-control, and cross-sectional studies, although those that were finally selected consisted of mostly prospective or retrospective observational studies.

### Exclusion Criteria

We excluded studies that were based only on adult populations, articles that were not written in English, review articles (used only for discussing their content), conference abstracts, and case reports (with one exception that illustrates the use of diabetic monitoring devices).

### Data Collection and Quality Determination for Individual Studies

For study identification and data collection, two analysts (SS and SAK) extracted and stored details of the underlying data in an excel database: These details were then used for analysis and scoring of the quality of the studies and quantitative synthesis later on. Appendix C lists sample data collection forms that were the basis for the collection of data in each study. Appendix D contains the evidence table for the 44 studies, which holds the collection of actual data from all 53 studies selected for quantitative synthesis (nine of these studies are previous reviews used for content discussion). Appendix E shows the meta-analysis for collaborative studies that involve multiple countries.

Several criteria were used to determine the quality of each study and the final score was used to classify the selected articles in this study as belonging to levels 1–4, where level-1 studies are of highest quality and level-4 is the lowest, as per the guidelines that are given by *AHRQ methods guide* (52, 53). Quality scores for individual studies are given in Appendix D in the evidence table. The following data and quality metrics were collected from each study. A combination of these scores was used to assign the final quality score (levels 1–4) to each study.

- **Study type**: Was it retrospective/prospective observational study or did it involve molecular work? A higher value was given for prospective studies compared to retrospective studies, while molecular studies were given the highest preference.
- **Study design:** Was the type of study regional/national/worldwide? was it a single/multi-center study? A higher value was given for national and/or multi-center studies.
- **Length of study:** Studies that lasted over a year had a higher value.
- **Patient characteristics:** studies with clearly stated details of participant numbers, age and gender were allocated a higher value. Studies with >1,000 participants, even distribution of age groups and equal representation of each gender were valued more.
- **Study quality:** Were outcomes predefined? Any presence of confounding elements/bias? Did the studies include many types of DM? Higher values were given for studies that had defined outcomes, had minimum bias and covered many types of DM included in the study.
- **Study standard:** Did the study have ethical approval, declaration of no conflict of interest (COI) by all authors and was the study funded? Higher values were assigned if the studies had all or at least any two of these standards satisfied.
- **Study outcome:** Did the study have a clear outcome/conclusion that matched its declared aim? Studies that satisfied these criteria were valued more.
- **DM occurrence:** Incidence and prevalence of DM (per 100,000 per year), if these figures were given. Not many studies gave these values clearly in their conclusions. Hence this information was not used to rank the studies, to maintain uniformity.

**Figure 2:**
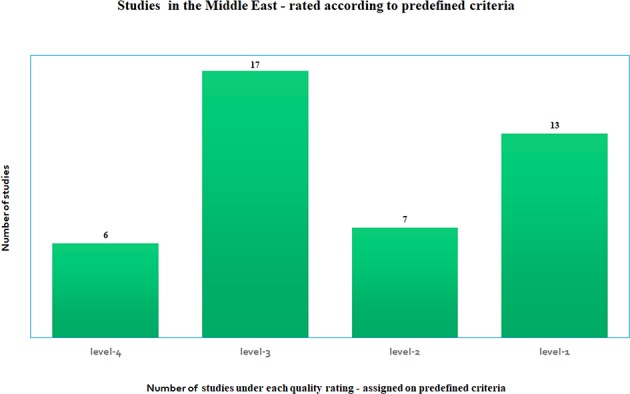
Number of studies under each quality rating—based on predefined quality criteria as satisfied by each study, where level-1 is the highest rating and level-4 is the lowest rating, where the rating is determined according to the *AHRQ methods guide* recommended standards for meta-analysis (52, 53).

The studies reviewed were widely varied in many of the criteria listed above such as the number of participants, length of study, type of study, outcomes, and disease covered. This variability did not make it meaningful to combine and compare them under one single criterion. Each study was scored under the common sets of criteria outlined above, as recommended by the *AHRQ methods guide* (52, 53), that *pertain to our study*. Level-1 is ranked as the highest rating and level-4 as the lowest rating. Figure 2 shows that there were 13 studies categorized as “level-1,” which essentially had two of three criteria such as ethics approval, COI declared and/or had funding, in addition to being long term studies with large cohort. There were seven studies that were classified as “level-2,” 17 studies which were labeled as “level-3” and six studies that did not meet most of the above criteria, were classified as “level-4.” Technology oriented studies were not ranked. Four Molecular studies were automatically assigned high values. If there were no known COI declared then the studies did not come up to a “level-1” grade (most of these studies were from previous years, from older studies). All studies were included in this review since missing information was not a criterion used to eliminate studies. These rankings were used to determine the degree of the contribution that each study made to our article as a whole.

### Data Synthesis and Analysis

For the methodological assessment, the following aspects were evaluated: Differentiation and classification between T1DM and T2DM, NDM, MODY, syndromic, autoimmune monogenic, insulin-dependent, estimation of prevalence and incidence.

## Results

### Type 1 Diabetes Mellitus in the Middle East

#### Studies Reporting on the Incidence and Prevalence of T1DM in the Middle East

The rates for childhood T1DM (prevalence and incidence) across the top ranked 10 countries is shown in Tables 2, 3 for the year 2015 for age <15 years and for the year 2017 for age <20 years (3, 4).

**Table 2:** Top 10 countries for children diagnosed with T1DM for age <15 years of age in 2015 and for age <20 in 2017 (3, 4, 55).

**(Rank) country/territory for <15 years**	**Number of pediatric patients diagnosed with T1DM in 2015 for age <15 years (3)**	**(Rank) country/territory for <20 years**	**Number of pediatric patients diagnosed with T1DM in 2017 for age <20 years (4)**
1. USA	84,100	1. USA	169,900
2. India	70,200	2. India	128,500
3. Brazil	30,900	3. Brazil	88,300
4. China	30,500	4. China	47,000
5. United Kingdom	19,800	5. Russian Federation	43,100
6. Russian Federation	18,500	6. Algeria	42,500
7. Saudi Arabia	16,200	7. United Kingdom	40,300
8. Germany	15,800	8. Saudi Arabia	35,000
9. Nigeria	14,400	9. ^*^Morocco	31,800
10. Mexico	13,500	10. Germany	28,600
**GLOBAL: NUMBER OF CHILDREN DIAGNOSED WITH T1DM**			
Global: number of children (<15 years) with T1DM	542,000	Global: number of children (<15 years) with T1DM	586,000
		Global: number of children (<20 years) with T1DM	1,106,200

*The numbers for 2017 are for a larger group of age <20, but the increase in numbers is much larger than the difference in the additional number of adolescents would bring, for years 15–20*.

* *The data for Morocco, extrapolated from Algeria*.

**Table 3:** Top 10 countries for children with incidence rates (per 100,000 per year) for T1DM, for age <15 years of age in 2015 and for age <20 in 2017 (3, 4, 55).

**(Rank) country/territory**	**Incidence of T1DM (per 100,000 per year) in 2015: for age <15 years (3)**	**(Rank) country/territory**	**Incidence of T1DM (per 100,000 per year) in 2017: for age <20 years (4)**
1. Finland	62.3	1. Finland	57.2
2. Sweden	43.2	2. Kuwait	44.5
3. Kuwait	37.1	3. Sweden	39.5
4. Norway	32.5	4. Saudi Arabia	33.5
5. Saudi Arabia	31.4	5. Norway	29.8
6. United Kingdom	28.2	6. Algeria	26.0
7. Ireland	26.8	7. Morocco^*^	26.0
8. Canada	25.9	8. United Kingdom	25.9
9. Denmark	25.1	9. Ireland	24.3
10. USA	23.7	10. Denmark	23.0
**GLOBAL: NEW CASES OF T1DM**			
Global: number of new cases of children (<15 years) with T1DM	86,000 (annual increase is 3%)	Global: number of children (<15 years) with T1DM	96,100
		Global: number of new cases of children (<20 years) with T1DM	132,600

**Figure 3:**
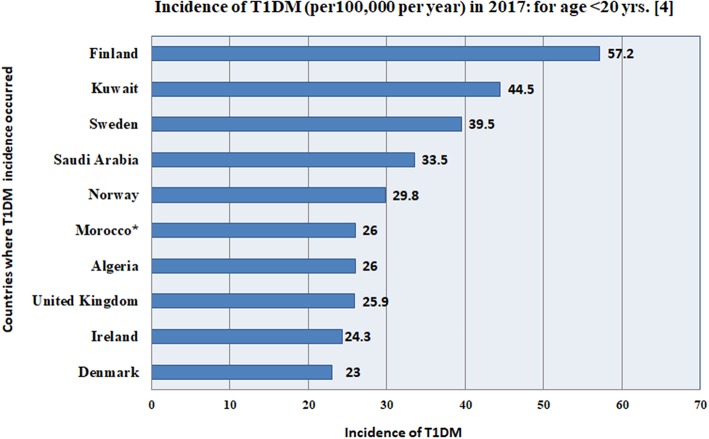
This figure gives the incidence (per 100,000 per year) for the top 10 countries in the world, according to the International Diabetes Federation (4), where, Kuwait and Saudi Arabia rank 2nd and 4th in the world with incidence rates of 44.5 and 33.5 per 100,000, for ages of children and adolescents <20 years. *The data for Morocco, was extrapolated from Algeria. The details for all 10 countries are shown in Table 3.

*The numbers for 2017 are for a larger group of age <20, but the increase in numbers is much larger than the difference in the additional number of adolescents would bring, for years 15–20. This data is illustrated in Figure 3*.

* *The data for Morocco, extrapolated from Algeria*.

The International Diabetes Federation has reported (4) that Saudi Arabia has one of the highest numbers (35,000) of children and adolescents (aged 0–19 years) with T1DM. Table 2 shows the numbers for the prevalence of T1DM in the top 10 countries, where Saudi Arabia's prevalence value increases from 16,200 for ages <15 years (ranked 7th in 2015) to 35,000 in 2017 for ages <20 years (4), although it falls by one rank to 8th place. Although the numbers for 2017 are for a larger group of age <20 (instead of age <15 years), the increase in numbers is much larger even after accounting for the additional number of adolescents this would bring for the years 15–20.

All values discussed below are incidence per 100,000/year. The rates for childhood T1DM across European countries vary between 40 and 67 for Sardinia (40), Sweden (47) and Finland (>60). This study found a higher incidence of T1DM occurring in males (1.3–2.0 times) compared to females, for children aged >15 years (55). The global trend has generally shown a steady increase in childhood-onset of T1DM while the age of onset is much earlier than seen before (55).

In a study conducted in Eastern Saudi Arabia, over a period of 18 years between 1990 and 2007, the average incidence rate for T1DM (438 patients, <15 years) rose from 18.05 in the first 9 years to 36.99 in the second half of the study, for an average increase of 27.52 per year (56). No significant increase in the incidence of T1DM was found in patients below 5 years of age (21% of the cohort) when compared to patients in the age group over 5 years of age. In a 5 years study (2004–2009) in North-West Saudi Arabia in Al-Madinah (57) on children below 12 years of age (419 patients) the mean age of onset was 6.9 ± 3.5 years, with an incidence rate of 29. This study found a higher incidence rate for children between 10 and 12 years of age, with the rate higher in girls (33) than in boys (22), but they did not find any significant annual increase in incident rates (57).

In a study conducted in the Al-Baha region in Saudi Arabia (58) over 10 years (2007–2016), on 471 children below 19 years of age, the prevalence rate of T1DM was dramatically high at 355, which could be cause for great concern. The female to male ratio in the cohort was 1:1.36 where T1DM was more common among girls at 57.5% compared to 42.5% among boys. This number is much higher than those given for other countries outside the Middle East (55).

The incidence of childhood T1DM varies from one country to another globally, as given for the top 10 countries in Figure 3 and Table 3. Kuwait and Saudi Arabia ranked 2nd and 4th in the world with incidence rates of 44.5 and 33.5 per 100,000 per year, for ages of children and adolescents < 20 years. Kuwait jumped from 3rd rank in 2015 to 2nd rank in 2017, while Saudi Arabia rose from rank 5 to rank 4 (although previous ranks in 2015 were for ages <15 years). The details for all countries are shown in Table 3.

Another study from the Eastern Province of Saudi Arabia found no etiological influences in children with new-onset T1DM for any of the following factors such as, maternal age at birth, birth order, birth weight, early introduction of cow's milk and cereals, infections and vaccines as well as nitrate levels in drinking water (59). These factors did not explain the rising incidence of T1DM in this population (59). Data on the national prevalence of T1DM, T2DM, and pre-diabetes in childhood is limited in Saudi Arabia. In one study, the incidence rate was calculated as 109.5 per 100,000 for Saudi Arabia and fifty adolescents and children were newly identified as having T1DM (60). The prevalence rate was highest at 243 per 100,000 for the age group 13–16 years of age, in this study. This study reports that the highest rate of prevalence for T1DM was 162 in the central region, and the lowest was 48 (per 100,000) in the eastern region (60).

The Saudi Abnormal Glucose Metabolism and Diabetes Impact Study (SAUDI-DM) was used to assess the prevalence of T1DM and T2DM, as well as impaired fasting glucose (IFG) among children and adolescents (61). Socioeconomic and demographic information, clinical details and measurements on common tests [weight, Body Mass Index (BMI), and height] were collected from randomly selected adolescents and children who were <18 years of age. The prevalence of diabetes in this cohort was 10.84 and 0.45% were known to have both T1DM and T2DM. Nearly 90% of the people in this study were not aware that they had diabetes. 10.39% of those in this study were newly diagnosed with either having diabetes (4.27%) or impaired fasting glucose (IFG) (6.12%). The statistics obtained from this study indicated that T1DM and T2DM were prevalent at a much higher rate than what was reported by international organizations and this included data on newly identified cases. There were many significant at-risk factors for developing DM and IFG which included data on age, gender, obesity, urbanization, higher income and the presence of lipids which are known to occur as a result of unhealthy diet and lifestyle (61).

Table 4 summarizes the statistics from the reviewed studies that report the incidence and prevalence of DM in Middle East countries such as Saudi Arabia, Kuwait, and Qatar. The incidence for Kuwait is 41.70 in 2017 for children <14 years of age (62). Qatar has lower prevalence at 28.39 for children between 6 months and 14 years of age when compared to Kuwait and the Kingdom of Saudi Arabia (KSA) which have higher prevalence at 39.5 and 29.8, respectively (Table 3). But, it still shows a 5.75% increase between the years 2012 and 2016 (Table 4) compared to 3–4% increase in Childhood T1DM worldwide (55) in Table 2. The rates for East Asian and Native Americans are very low at 0.1 and 8, which are nowhere near the higher rates for all other countries. Perhaps a comparative study that includes differences in the genome, food habits and environmental factors between these countries and those in the Middle East can help to identify causative factors that can help with DM management.

**Table 4:** Statistics from the reviewed studies on the occurrence of T1DM in the Middle East.

**Disease-country**	**Study period**	**Age (year)**	**Study type and design**	**Study size**	**Incidence (per 100,000)**	**References**
Kingdom of Saudi Arabia (KSA)	(1990–1998) to (1999–2010)	<19	Observational, case	119	18.05–36.99[Table-fn TN3]	(3, 4, 56, 57, 59)
KSA Nationwide	2001–2007	<19	Survey, case	45,682	48–162^#^	(60)
KSA	2010	<15	Observational, case	438	27.5–36.99	(56, 57)
KSA	2018	<19	Observational, case	471	355	(58)
Kuwait	2017	<14	Observational, case	515 (247 boys, 268 girls)	39.3 (boys) 41.70 (girls)	bib62
Qatar (T1DM)	2006–2011	<14	Prospective, case	440	23.15	(47)
Qatar (T1DM)	2012–2016	<14	Prospective, case	440	28.39[Table-fn TN3]	(47)
Qatar T2DM	2012	5 < age <14	Prospective. Case	45	1.82[Table-fn TN5]	(47)
Qatar T2DM	2012–2011	5 < age <14	Prospective, case	45	2.7–2.9[Table-fn TN3]	(47)
Iran	2000–2015	<15	Observational, case	988	13.35[Table-fn TN6]	(49)

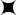
*Unadjusted rate. This is an increase of 5.24 since 2011, with 90% CI of 31.82–40.03*.

# *On an average the incidence rate was 109.5*.

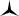
*With no incidence prior to 2008 recorded*.

^
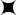
^*A doubling in incidence of 18.94 between the years 1998 and 2010 and ^
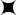
^an increase of 0.88 since 2012*.

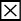
*Rapid rise from 89 to 134 to 691 new cases*.

A study from Kuwait reported the incidence of childhood-onset T1DM during the years 2011–2013 for children who were below age 14 (47, 62) and compared it with a previous study done in 1992–1997. This study detected an increasing trend in the incidence of T1DM from 17.7 in the previous study to 40.9 per 100,000 per year in 2011–2013 (2.3 times higher), as detailed in Table 4 (62). They found a higher incidence rate for girls (44.1) when compared to boys (39.3) (47, 62). Another study from Kuwait aimed to understand the social and metabolic characteristics [lipids, lipoproteins, apolipoproteins, lipoprotein (a), and total sialic acid] and predisposing factors in 6–18-year old Kuwaiti children with T1DM (63). Children's metabolic and social characteristics were affected negatively when compared to those who were normal controls (63).

A prospective cohort study was performed in Qatar to estimate the occurrence of T1DM and T2DM among patients who were below 14 years (47). The aim was to ascertain all new cases of T1DM and T2DM in Qatar, in the only tertiary care center treating children with DM in Qatar. The results, given in Table 4, indicate there is an increase in the incidence rates of T1DM and T2DM between the years 2006 and 2016 (47).

Another study from Qatar compared the difference between familial T1DM and non-familial T1DM in terms of the clinical aspects and other biochemical measures such as lab results. This retrospective study, conducted between 2012 and 2016, across a cohort of children and youth with T1DM (*n* = 424), aged between 6 months and 16 years, concluded that familial T1DM was more prevalent in boys than girls (1.4:1, respectively). The prevalence of non-familial T1DM (1:1.1, respectively) did not differ between genders (64). Familial T1DM occurred relatively early in childhood (40.7% before the age of 4 years and 72% before 9 years of age) vs. non-familial T1DM which occurred relatively later in life (80% when they are over 4 years old and 40% after they were 9 years or older). Familial T1DM was more prevalent in boys vs. girls and occurred earlier in childhood compared to non-familial T1DM (64).

A recent study from Iran also found that the annual incidence of T1DM for children under 15 years of age, between the years 2000 and 2015, was 13.35/100,000 (49). Adding the 2nd study period (15 years in total), the study disclosed a rapid rise of incidence as 89, 134, and 691 new diabetes cases for the 1st, 2nd and 3rd years respectively, over a period of 5 years (where new cases were at the rate of 5 per year) (49).

Table 5 reports on country-wise statistics on the occurrence of T1DM in the Middle East from IDF Diabetes Atlas: Country reports 8th Edition (4).

**Table 5:** Country-wise statistics on the occurrence of T1DM in the Middle East from IDF Diabetes Atlas: Country reports 8th Edition (4).

**Country**	**Year**	**Age**	**# of children and adolescents with T1DM; (# of newly diagnosed children and adolescents in 2017, per 100,000)**
KSA	2017	<19	34,981; (33.5)
Kuwait	2017	<19	5,496; (44.5)
Qatar	2017	<19	592; (12.2)
Iran	2017	<19	9,009; (4.0)
Oman	2017	<19	355; (2.7)
Bahrain	2017	<19	96; (2.7)

#### Studies Reporting on the Autoantibody Status in the Middle East

There is limited data on autoantibody status in T1DM in the Middle East. A study was conducted on patients diagnosed with T1DM and T2DM to determine the prevalence of auto-antibodies GAD65 (GADA) and IA-2 antibodies (IA-2A) of Saudi diabetic patients living in Jeddah (65). Eight out of 99 patients who had T2DM tested positive for GADA and three of these patients (who had the disease for a shorter time) were also positive for IA-2A. Here, the association of these autoantibodies was in patients who had an early-onset of T2DM, where GADA was positive in 54% of T1DM and IA-2A was positive in 27%. All patients who had T2DM and who tested autoantibody-positive were treated with insulin therapy (65). In the Middle East, autoantibodies are likely to be found if the disease-onset is at a younger age (65). Female patients with T1DM were more likely to have GADA present (65).

The study from Qatar **(**as given in Table 6) (66) reviewed all clinical and biochemical data, including beta-cell autoimmunity (GADA, ICA, and IAA) over 5 years. These values were analyzed and the results were compared with other studies to measure the prevalence of autoantibodies and their relationship to related diseases. This study reports a higher rate of T1DM occurrence for Qatar compared to the other countries and they reported that these incidences increased over the study period. They also report a higher prevalence of diseases related to the autoimmune abnormalities as shown in Table 6 and recommend regular screening of patients for these disorders (66). It can be seen from the values in this table that a larger percentage of the T1DM patients have severe autoimmune response compared to the T2DM group, for each of the categories, except for Anti-insulin Ab, where T2DM percentage for prevalence is higher.

**Table 6:** Results of a cross-sectional study conducted in Qatar, on the antibody status in 490 T1DM and T2DM patients during 2012–2016 (66).

**Antibody status in T1DM and T2DM (Age 0.5–16 years) over 5 years—2012–2016**		
**β-cell autoimmunity**	**TIDM (431 patients)**	**T2DM (59 patients)**
Anti-GAD (Anti-glutamic acid decarboxylase)	75.5	29.3
Anti β-islet (Ab) (antibody)	53.4	29.4
Anti-insulin Ab (antibody)	40.4	58.3
All 3 antibodies listed above, together in a patient	18.4	No one
**Thyroid function**	**TIDM**	**T2DM**
Hypothyroidism (FT4 <11.5 pmol/L)	10.6	10.0
Subclinical hypothyroidism	3.5	8
High TPO with normal thyroid function	22.7	23.1
High anti TPO	27.2	34.6
ATT IgA	5	8.7
ATT IgG	4.4	Not detected
Celiac Disease in ATT IgA and IgG positive patients	75% of patients (9/12)	

#### Studies Reporting on the HLA Haplotypes Among the Middle East Populations

Table 7 shows the heterogeneity in HLC class II haplotype distribution found among Lebanese and Bahrainis. This table lists the alleles, haplotypes, differing associations, and frequency of homozygous alleles (67). The results of this study, indicate that, when determining a patient's susceptibility to T1DM, with respect to a specific HLA haplotype, the patient's ethnic and racial background needs to be taken into consideration.

**Table 7:** HLA class II haplotypes distribution among Bahraini and Lebanese T1DM patients (67).

**Heterogeneity in HLA class II haplotypes in T1DM patients**		
**Types**	**Bahraini-alleles and haplotypes (252 subjects)**	**Lebanese-alleles and haplotypes (189 subjects)**
**SUSCEPTIBLE ALLELES AND HAPLOTYPES**		
Susceptible Alleles-shared	DRB1^*^030101, DQB1^*^0201	DRB1^*^030101, DQB1^*^0201
Susceptible Alleles	DRB1^*^040101	DRB1^*^130701
Susceptible Haplotype-shared	DRB1^*^030101-DQB1^*^0201	DRB1^*^030101-DQB1^*^0201
**PROTECTIVE ALLELES**		
Protective Alleles-shared	DRB1^*^100101, DQB1^*^030101	DRB1^*^100101, DQB1^*^030101
Protective Alleles		DQB1^*^050101
Protective Haplotypes-shared	DRB1^*^070101-DQB1^*^0201 and DRB1^*^110101-DQB1^*^030101	DRB1^*^070101-DQB1^*^0201 and DRB1^*^110101-DQB1^*^030101
**DIFFERENTLY ASSOCIATED**		
Susceptible or neutral	DRB1^*^040101-DQB1^*^0302 and DRB1^*^040101-DQB1^*^050101	
Protective		DRB1^*^040101-DQB1^*^0302 and DRB1^*^040101-DQB1^*^050101
**THE FREQUENCY OF HOMOZYGOUS ALLELES**		
Higher	DRB1^*^03011-DQB1^*^0201	
Higher		DRB1^*^110101-DQB1^*^030101
**GENOTYPES**		
Major genotype	DRB1^*^030101-DQB1^*^0201/DRB1^*^040101-DQB1^*^0201	
Less frequent genotype		DRB1^*^030101-DQB1^*^0201/DRB1^*^040101-DQB1^*^0201

T1DM patients in Bahrain have similar associations between DRB1 and DQB1 alleles and diabetes as was found in European populations (who may or may not have Arab descent), such as individuals in Turkey (68), Spain (69), and the United Kingdom (70). These data suggest that diabetes that occurs in children below 5 years of age indicates a high familial risk (70). On the contrary, there was only a weak association between DRB1^*^040101-DQB1^*^0302 with T1DM in the Bahraini population and there was no negative association with DRB1^*^1501-DQB1^*^06 with T1DM (71).

#### Studies Reporting on Diabetes Complications

It is estimated that around 96,000 children who are <15 years old will develop T1DM every year and between 13 and 80% of these children are expected to have DKA when they are diagnosed with T1DM. The highest number of cases were found in the UAE, KSA, and Romania and the lowest occurrences were in Canada, Sweden, and the Slovak Republic (48). The frequency of DKA is significantly greater in T1DM adolescents with a higher HbA1c level, lipodystrophy and those who had discontinued insulin treatment (72). Most of the studies relating to DKA in the Middle East are from Saudi Arabia. The UAE and Saudi Arabia have the highest frequencies (80 and 44.9% respectively) of DKA in children at the time of presentation (73).

Infections were the most common precipitating factor for DKA (82.1%) in Al-Baha, Saudi Arabia (74). An episode of DKA was the first clinical presentation of diabetes among 52 (65%) patients (74). In a retrospective study from a single center in Saudi Arabia, the predominant precipitating cause of DKA were viral infections and non-compliance to the insulin regimen of the diagnosed diabetic cases (75). In Al-Madina, Saudi Arabia, DKA affected 55.3% of the patients on disease-onset, where the average age of the patients was 6.7 years and the female: male ratio was 1.4: 1 (76).

In a study from Kuwait of all children diagnosed with T1DM, 36.7% had DKA with young children (0–4 years) at the highest risk (77). Data which was obtained from the Eastern province of Saudi Arabia shows that three-quarters of patients with T1DM had ketoacidosis on presentation (78). In a study from the Pediatric Endocrinology Clinic of the Maternity and Children Hospital, Jeddah, from 2000 to 2014, the most significant independent predictors of DKA were poor compliance with a healthy lifestyle and an excess intake of sweets (79).

### Studies Reporting on the Incidence and Prevalence of T2DM

A retrospective cross-sectional study addressed the prevalence of hyperinsulinism and T2DM in overweight and obese Saudi children (80). The overall prevalence of T2DM was 9.04% (80). Among children and adolescents with T2DM, the majority (62.86%) had a body mass index (BMI) ≥ 85th percentile, 37.14% had a BMI ≥ 95th percentile (80).

A retrospective study from the Al-Ain hospital from UAE characterized the features of T2DM among children and adolescents. Of 96 young people newly diagnosed with DM, 11% were identified as having T2DM (81). The clinical characteristics were: pubertal onset, female preponderance, obesity, strong family history of T2DM, high plasma glucose at presentation, adequate beta-cell reserve and antibody negativity.

A study from Kuwait determined the prevalence of T2DM among patients between the ages of 6–18 years. Children with T2DM were identified at 182 schools (50 primaries, 63 intermediate, and 69 secondaries), randomly selected, using the 2000/2001 educational districts' registers as a sampling frame. T2DM was identified in 45 of the 128,918 children surveyed, thereby giving an overall prevalence of 34.9 per 100,000, with significantly different prevalence for males at (47.3, 95%) compared to females (26.3, 95%), with a trend for increased prevalence with age (*p* = 0.026). The final age-adjusted prevalence values for the Kuwaiti population for T2DM, in the year 2002, was 33.2, 41.6, and 24.6 for overall, male and female groups, respectively (82).

GWAS studies have successfully identified over 80 variants found in T2DM patients with small effect size where the risk for T2DM diabetes increased between 5 and 40%. A majority of these genes regulate insulin secretion while a few regulate insulin sensitivity (83). But, a recent study has indicated that T2DM variants (rs7903146 and rs4506565) in Asian and European populations are not predictors of T2DM in the Qatari population (84). Hence this study suggests that Qatari population might have different variants that might be risk factors for T2DM in this region.

### Studies Reporting on the Incidence and Causes of Neonatal Diabetes Mellitus

Several studies conducted in the Gulf region have reported higher incidences of NDM compared to worldwide estimates reaching 1:260,000 live births (85). Incidence of PNDM is 1:31,900 in the United Arab Emirates (UAE) (86), and 1 in 21,196 live births in KSA (85), mainly as part of rare autosomal recessive syndromes. A study from Iran found that Wolcott-Rallison syndrome was a common cause of PNDM (87). In a study published in Oman, genetic abnormalities were found in 15/24 (62.5%) of their patients with PNDM (88).

### Maturity Onset Diabetes of the Young

The incidence of monogenic forms of diabetes in childhood has not been identified in Saudi Arabia or any of the Middle East countries (89).

### Studies Reporting on Syndromic Forms of Diabetes Mellitus

Autosomal recessive syndromic disorders that are generally considered rare, are highly prevalent in the Arabian Gulf region. The highest incidences worldwide were reported from Saudi Arabia in association with PNDM (85). Fourteen out of 17 (82.4%) patients had been affected due to inheriting defective genes that cause Wolcott-Rallison syndrome (41%), NDM and hypothyroidism (29.4%), Fanconi-Bickel syndrome (5.8%), and thiamine-responsive megaloblastic anemia (5.8%). Another study from Emirates reported nine out of 25 patients with PNDM (36%) with Wolcott-Rallison Syndrome (86).

### Mitochondrial Diabetes Mellitus

No studies have reported the incidences of childhood mitochondrial DM in the Middle East region.

### Miscellaneous Forms of Diabetes Mellitus

Very little data is available on the prevalence of mutations in FOXP3, AIRE, SIRT1, and STAT3 in the Middle East, except for a few case-reports from children in the Arabian Gulf region (90).

### Studies Reporting on the Use of Technology to Improve Diabetes Management

#### Added Value in Using Insulin Pumps

A study was conducted in UAE to see if there can be better health perception and patient satisfaction after treatment, if insulin pumps were used by children and adolescents (91). The authors found that it provides more value in terms of treatment and satisfaction, irrespective of the duration of use of the insulin pumps.

#### Added Value in Using Monitoring Devices That Aid Insulin Control

Another study from the UAE investigated various insulin pump functions and their efficacy in controlling blood glucose. CareLink® Pro 3 software was used for 8–12 weeks (92). They found that if the patient combines the use of Bolus wizard with frequent blood glycemic monitoring, it could help to control blood sugar levels. Another study assessed the benefits of using the flash glucose monitoring system (FGMS) in children and adolescents with T1DM during Ramadan fasting (93). They found that this device could help to fast during Ramadan, without being subject to life-threatening situations that might arise due to hypoglycemia or DKA. Another study from Riyadh, Saudi Arabia, conducted a prospective pilot study of 51 participants with T1DM where they compared the use of the flash glucose monitoring system (FGMS) against the use of continuous subcutaneous insulin infusion (CSII). They did not find much difference among users during Ramadan fasting but found that CSII helped to keep the fluctuations in glucose levels to a minimum (94).

In a study from Qatar, CSII significantly improved glucose control in T1DM children and adolescents who use a standardized protocol. A reduction of HbA1c by 1.6% was achieved after 1 year of CSII initiation (95). A report from Qatar was the first to demonstrate the use of the hybrid closed-loop system in managing a patient with T1DM that resulted in a 1.3% decrease in HBA1c value. The time in range significantly increased to 77% with sensor glucose (SG) values of 139 ± 60 mg/dl, sensor wear of 82% and an auto mode period of 84% per week (96), suggesting that immediate adjustment of the bolus wizard settings such as the ICHR, ISF, and active insulin time should be considered.

## Discussion

Childhood DM is a health problem with major health implications in all regions in the Middle East. This review has highlighted the high incidence/prevalence of different types of childhood DM in this region that include NDM, T1DM, T2DM, and syndromic forms of DM. The high incidence of childhood DM in this region imposes a large economic and social burden on the population. We looked at different regions in the Middle East, where the children's population has been affected by DM and its various manifestations. We also discuss the type of studies that we reviewed and discuss the limitations of this study in terms of selection and language bias. Finally, we answer some of the questions for which this study sought answers, in section Aims of this Study.

Our review of the literature suggests that most of the research reported from the Middle East on childhood DM relates to a large number of a retrospective reviews of notes, a few prospective case studies, systemic reviews of the literature, questionnaire-based studies and case reports. A significant number of retrospective studies report on the incidence and prevalence of different types of DM in childhood in the Middle East and as with all retrospective studies these have the potential to be affected (to some degree) by confounding factors and bias. A few prospective studies have reported on the incidence and prevalence of T1DM.

The results of our systemic review did not find any research studies reporting on the establishment of National Childhood Diabetes Registries in any of the Middle Eastern countries. There is a lack of studies focusing on national epidemiology data of different types (such as NDM, T2DM, T2DM, MODY, and syndromic forms) of childhood DM, limited studies on the full complement of autoantibody status (GAD65, Islet, Insulin, and Zinc autoantibodies) in T1DM and HLA haplotype of different populations in the region. Only a few studies report the use of modern technological advances in the management of DM in childhood from the Middle East. Apart from a few research studies in NDM, there is a lack of studies which address the understanding of the molecular basis of rare forms of DM (which are so prevalent in this part of the world) and developing novel therapies or undertaking clinical trials for common or for these rare forms of childhood DM. Finally, it is unclear from our systemic review if there are any national or regional research funding organizations for childhood DM.

National registries hold collective information on diseases of national interest that can be used to plan and regulate healthcare delivery to the population. Childhood DM is one of the major health problems in the Middle East and yet there is no established National Children's Diabetes registry in any of the countries in this region. These registries can influence and improve health outcomes and reduce health care costs. The information in these registries can be used to competitive advantage by the healthcare providers by adopting best practices. Therefore, establishing National Childhood Diabetes registries is pivotal to the Middle East Region, to advance research and ensure continued health care delivery to the highest standards. National Diabetes registries have been successfully implemented in developed countries such as the United States, Australia, and England (97). Interdisciplinary efforts by registries in these countries, where data is obtained from multiple sources such as physicians, regulatory bodies, national health centers, and other care providers have helped to control and manage the disease and reduce socioeconomic costs (98).

The high prevalence of several different types of childhood diabetes including T1DM, T2DM, NDM, and syndromic forms of DM, provides a unique opportunity to develop research collaborations between the different Middle East countries. However, in our review, there were very few collaborative research studies between the different countries in this region. Government or public health organizations can play a key role in funding and promoting health care programs that will help to reduce the occurrences of chronic illnesses such as the different types of childhood DM. One such implementation program by the national center for chronic disease prevention and the centers for disease control and prevention has helped patients to manage their illnesses better (99). As T1DM is becoming so prevalent in the Middle East the establishment of a reference biochemical/immunology laboratory for measuring diabetes antibodies should be prioritized.

There is very little knowledge that relates to childhood diabetes research-funding opportunities in the Middle East as this information is not freely available. No formal joint funding organizations between different countries have been established which could fund childhood diabetes research in the Middle East. There is a dire need to establish collaborative research funding opportunities for childhood diabetes research in this region. Traditionally funding for registries has been sourced from various stakeholders who might be interested in sharing the data collected, such as foundations interested in the history, progress and therapeutic options available for diabetes, government, insurance and regulatory bodies who are interested in the long-term effects and results of traditional and optional treatments, pharmaceutical and device manufacturing companies, patient groups, private funding, and professional societies. Proactively contacting these institutions or resonding to their request for proposals (RFP) might lead to the discovery of unmet needs that can fulfill the funding requirements (100).

Organizations such as the Diabetes UK, the Juvenile Diabetes Research Foundation (JDRF) provide project grants that support high-quality basic and translational research work on the causes and treatment of diabetes. These funds help to make sure that research is progressive, proper and timely treatments are delivered to the families affected by diabetes and these families are supported and given a voice. The American Diabetes Association works with government and health administrative offices to ensure that enough resources are allocated for diabetes research. Similar funding organizations that are geared to support diabetes studies targeted to the local populations can be set up in the Middle East region.

The Middle East region has an abundant resource of patients with rare and unusual forms (for example NDM and syndromic forms of DM) of childhood DM. Patients with NDM and syndromic forms of DM are rare in the Western world but relatively common in the Middle East region. For example, Saudi Arabia and the UAE have the highest incidences of NDM anywhere in the world. This rich resource of unique patients provides an unprecedented opportunity for undertaking molecular biology research in childhood DM and developing novel therapies for these rare conditions in this region of the world. Understanding the molecular mechanisms of DM in these patients provides fundamental new insights into normal physiological mechanisms involved in the development of DM in the childhood period and for novel disease discovery. More importantly, having a genetic basis for diagnosis can greatly change patient management (for example in some cases of NDM or MODY diabetes).

**Figure 4:**
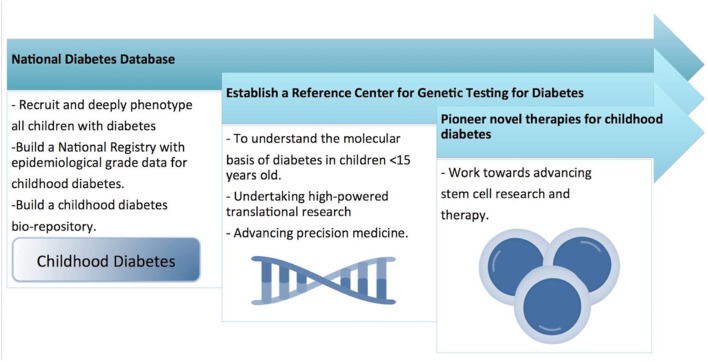
Shows the national research strategy for childhood diabetes, for developing a national wide pediatric diabetes surveillance and intervention system.

However, in our review, we were struck by the lack of studies in the Middle East region which focus primarily on understanding the molecular mechanisms of the different forms of childhood DM. Several studies have reported the molecular mechanisms of some types of DM (such as NDM) but the molecular analysis was performed by collaborating with laboratories outside the Middle East region and involved sending blood or DNA samples for analysis to laboratories outside of the region. To address this issue, we suggest that a regional molecular genetics laboratory needs to be established which will serve the needs of all the countries in the Middle East for genetic testing for all forms of childhood DM. A pipeline system should be implemented so that all clinicians can send blood or DNA samples for processing to this regional molecular genetics laboratory (Figure 4). This will allow the establishment of a Middle East centralized database and patient registry for all children who are genetically tested for DM.

### Lack of Funding Specifically for Pediatric Diabetes Studies

A random sample of 10,501 outcomes reported in the Qatar National Research Fund (QNRF) website (as of February 28th 2019) was extracted. This list consisted of publications or articles (online, journal and conference papers, book chapters, creative work, public report, and patents) that were reported as products of 1,223 unique grant awards over the past years. Of these publications, only 89 grants have the keyword “diabetes” in their title, but none of these 89 titles include the keyword “pediatric.” There were only five titles among the list of 1,223 grants, which had the word “pediatric,” but none of these studies were related to diabetes. Hence it is reasonable to estimate that there is no specific funding specifically for pediatric diabetic studies and there might be very few exceptions in more recent years, for which publications are yet to be reported under the grants.

## Limitations of This Study

This study has several limitations. Firstly, we were not able to establish if there are any local or regional organizations (like Diabetes UK or JDRF) which traditionally dedicate funding for childhood diabetes research in the Middle East. This information was not easily accessible anywhere. It is possible that there are childhood diabetes research funding institutions in the different Middle Eastern countries but we were not able to capture this information. Secondly, we excluded case reports (with one exception) as a measure of research activity and there were a large number of cases published on patients from the Middle East region so this may well be underreported and introduce bias in our analysis. Thirdly there were some publications that report diabetes research outcome measures in both children and adults together. These were again excluded in the final analysis and could represent a source of bias.

### Limitations Due to Selection Bias in Using Only English Language Articles

A meta-analysis of 303 studies, has been conducted by Juni et al. (101) to estimate the effect of language bias introduced due to the selection of only English language articles and omission of other language articles published in local journals. Their study found that non-English language articles on trials had a lower number of participants and in some cases reported more significant results. In addition, the quality of the methodology in non-English reviews was lesser than it was in English language articles. In some studies, treatment effects were generally shown to be higher in non-English language publications but were shown to be lesser in other publications. This study concluded that there might be very little difference (as little as 5%) in estimates of treatment effects that were published in English vs. non-English language trials, while there were mixed benefits shown in other studies that were not conclusive. In another study conducted by Egger et al. (102) the authors found that study authors chose English language journals when the results of their studies were more statistically significant [with an odds ratio of 3.75 (95% CI 1.25–11.3)] than when the results were less significant. So, although we recognize that there might be a bias in our review by including only English language articles, there is no clear evidence one way or the other that establishes definite bias if authors select only English language articles. Hence, we opted to include only English language articles.

### Limitations Due to Bias in the Selection of Types of Studies

**Figure 5:**
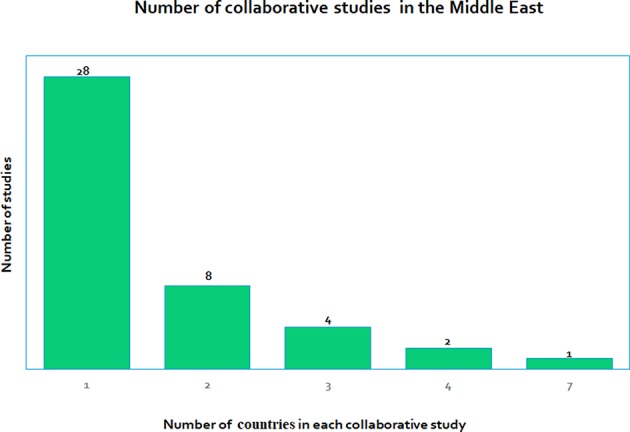
Shows the number of collaborative research studies in the Middle East, with studies involving only one country (28 studies) to some that involve many countries, the highest being seven countries in one of the studies.

Many of the studies we reviewed were retrospective or prospective observational studies and not interventional or translational. We found that an overwhelmingly large number of papers were publications that were related to non-Arabic or mixed Arabic cohorts related to DM. Most studies were based only on adult populations and comparatively lesser publications were centered on the pediatric populations, which could introduce some population bias in the information obtained. Many of the 43 studies were clinical with only four studies that were considered to be molecular. There were only three studies related to technology. Hence there could be bias in the selection of the type of study due to the non-availability of literature in terms of molecular studies and technology evaluating studies. We also found that there were not many collaborative studies where more than one country was involved. Figure 5 gives the number of collaborations for each study, which ranges from single country studies to one that has up to seven countries involved in a study.

## Key Questions That Were Answered in This Study

- What types (basic, clinical, and translational) of research has been reported in childhood DM? There were no basic or translational research studies. Mostly there were clinical studies, with a few molecular and technological studies.
- What impact does this research have on the local population of children in the Middle East? These studies were very relevant to the local population but the emphasis was on the management of the disease rather than on offering better and improved treatment options.
- What research strategies are in place to tackle the burden of childhood DM in the Middle East? An increasing number of molecular studies are taking place that identifies factors pertaining to the local population. This can improve the standard of health care for the local population.
- What funding opportunities are available for childhood DM research in the Middle East? Only six of the 44 projects were funded in this review. Increasing funding opportunities for childhood DM research is imperative.
- What collaborations exist between different Middle Eastern countries in childhood DM research? We found that most studies (28) were stand-alone and performed in one country only, whereas there were other studies where several countries were involved (Figure 5), where the number of collaborating countries ranges from 2 to 7.

## Conclusions and Recommendations

Childhood DM is a major health burden for the Middle East region which needs to be addressed urgently. The incidences of both T1DM and T2DM in childhood are increasing rapidly in the Middle East region and urgent research efforts are needed to be focus on understanding the reasons behind this. Comprehensive national and regional epidemiological data on all types of childhood DM needs to be collected and databases set up. National and regional funding schemes for basic and translational childhood diabetes research should be established with support from central governments. The challenges of childhood DM can only be tackled by undertaking focused research which addresses the issues of regional collaboration, establishing a regional molecular genetics laboratory, building comprehensive epidemiology data, focusing on understanding disease mechanism/s and pathophysiology and establishing a regional childhood diabetes research funding organization. As the field of childhood DM advances and new treatments come on board, the Middle East region should be prepared to embrace and implement the new state of the art research [such as possible islet transplantation, stem cell-based therapies, induced pluripotent stem cell (iPSC) based treatments and immunomodulation therapies] that will benefit patients and undertake clinical trials of potential new therapies for childhood DM. The rich resources of the Middle East need to be channeled so that all children with DM in the Middle East will benefit from such translational research. A new generation of scientists, beta-cell physiologists, epidemiologists, diabetologists, and physicians looking after children with DM need to engage, collaborate, and develop a strategic vision so that they can make strides in this extremely important research area. These initiatives have the potential to manage the burden of childhood DM in the Middle East and improve the quality of lives of all children with diabetes. Investing in and funding basic and translational childhood diabetes research will bring enormous benefits financially, economically, and socially for the whole of the Middle East region.

## Data Availability Statement

All datasets generated for this study are included in the article/Supplementary Material.

## Author Contributions

SS and SA-K carried out the search and screened the titles and abstracts to retrieve papers. KH and NE selected articles of interest. All authors were involved in the writing and editing of the manuscript.

### Conflict of Interest

The authors declare that the research was conducted in the absence of any commercial or financial relationships that could be construed as a potential conflict of interest.

## References

[1] Organization WH Global Health Estimates 2016: Disease burden by Cause, Age, Sex, by Country and by Region, 2000–2016. Geneva: World Health Organization (2018).

[2] Organization WH Global Health Observatory (GHO) Data Map Gallery (2017). Available online at: https://www.who.int/uat-portal/map-gallery

[3] Federation ID IDF Diabetes Atlas, 7th Edn. Brussels: International Diabetes Federation (2015).

[4] Federation ID IDF Diabetes Atlas: 8th Edn. Brussels: International Diabetes Federation (2017).

[5] SEARCH for Diabetes in Youth Study Group Liese AD D'Agostino RB Jr Hamman RF Kilgo PD Lawrence JM. The burden of diabetes mellitus among US youth: prevalence estimates from the SEARCH for Diabetes in Youth Study. Pediatrics. (2006) 118:1510–8. 10.1542/peds.2006-0690 17015542

[6] Peraldo M Fasulo A Chiappini E Milio C Marianelli L. Evaluation of glucose tolerance and insulin secretion in cystic fibrosis patients. Hormone Res. (1998) 49:65–71. 10.1159/000023128 9485173

[7] Banjar H Angyalosi G. The road for survival improvement of cystic fibrosis patients in Arab countries. Int J Pediatrics Adolesc Med. (2015) 2:47–58. 10.1016/j.ijpam.2015.05.006 30805437 PMC6372404

[8] Patterson CC Dahlquist GG Gyurus E Green A Soltesz G Group ES. Incidence trends for childhood type 1 diabetes in Europe during 1989–2003 and predicted new cases 2005–20: a multicentre prospective registration study. Lancet. (2009) 373:2027–33. 10.1016/S0140-6736(09)60568-7 19481249

[9] Ehehalt S Dietz K Willasch AM Neu A Baden-Wurttemberg Diabetes Incidence Registry Group. Epidemiological perspectives on type 1 diabetes in childhood and adolescence in germany: 20 years of the Baden-wurttemberg Diabetes Incidence Registry (DIARY). Diabetes Care. (2010) 33:338–40. 10.2337/dc09-1503 19903753 PMC2809277

[10] Undlien DE Kockum I Ronningen KS Lowe R Saanjeevi CB Graham J. HLA associations in type 1 diabetes among patients not carrying high-risk DR3-DQ2 or DR4-DQ8 haplotypes. Tissue Antigens. (1999) 54:543–51. 10.1034/j.1399-0039.1999.540602.x 10674967

[11] Eisenbarth GS. Type I diabetes mellitus. A chronic autoimmune disease. N Engl J Med. (1986) 314:1360–8. 10.1056/NEJM198605223142106 3517648

[12] Ziegler AG Rewers M Simell O Simell T Lempainen J Steck A. Seroconversion to multiple islet autoantibodies and risk of progression to diabetes in children. JAMA. (2013) 309:2473–9. 10.1001/jama.2013.6285 23780460 PMC4878912

[13] Towns R Pietropaolo M. GAD65 autoantibodies and its role as biomarker of Type 1 diabetes and Latent Autoimmune Diabetes in Adults (LADA). Drugs Future. (2011) 36:847. 10.1358/dof.2011.036.11.1710754 22869930 PMC3411186

[14] Shivaprasad C Mittal R Dharmalingam M Kumar PK. Zinc transporter-8 autoantibodies can replace IA-2 autoantibodies as a serological marker for juvenile onset type 1 diabetes in India. Indian J Endocrinol Metab. (2014) 18:345–9. 10.4103/2230-8210.131174 24944929 PMC4056133

[15] Delvecchio M Mozzillo E Salzano G Iafusco D Frontino G Patera PI. Monogenic diabetes accounts for 6.3% of cases referred to 15 Italian pediatric diabetes centers during 2007 to 2012. J Clin Endocrinol Metab. (2017) 102:1826–34. 10.1210/jc.2016-2490 28323911

[16] Karalliedde J Gnudi L. Diabetes mellitus, a complex and heterogeneous disease, and the role of insulin resistance as a determinant of diabetic kidney disease. Nephrol Dial Transplant. (2014) 31:206–13. 10.1093/ndt/gfu405 25550448

[17] Robert AA Al Dawish MA Braham R Musallam MA Al Hayek AA Al Kahtany NH. Type 2 diabetes mellitus in saudi arabia: major challenges and possible solutions. Curr Diabetes Rev. (2017) 13:59–64. 10.2174/1573399812666160126142605 26813972

[18] Hruby A Hu FB. The epidemiology of obesity: a big picture. Pharmacoeconomics. (2015) 33:673–89. 10.1007/s40273-014-0243-x 25471927 PMC4859313

[19] Schwitzgebel VM. Many faces of monogenic diabetes. J Diabetes Investig. (2014) 5:121–33. 10.1111/jdi.12197 24843749 PMC4023572

[20] Johansson BB Irgens HU Molnes J Sztromwasser P Aukrust I Juliusson PB. Targeted next-generation sequencing reveals MODY in up to 6.5% of antibody-negative diabetes cases listed in the Norwegian Childhood Diabetes Registry. Diabetologia. (2017) 60:625–35. 10.1007/s00125-016-4167-1 27913849

[21] Molven A Njolstad PR. Role of molecular genetics in transforming diagnosis of diabetes mellitus. Expert Rev Mol Diagn. (2011) 11:313–20. 10.1586/erm.10.123 21463240

[22] Slingerland AS Shields BM Flanagan SE Bruining GJ Noordam K Gach A. Referral rates for diagnostic testing support an incidence of permanent neonatal diabetes in three European countries of at least 1 in 260,000 live births. Diabetologia. (2009) 52:1683–5. 10.1007/s00125-009-1416-6 19499210 PMC2709852

[23] Iafusco D Massa O Pasquino B Colombo C Iughetti L Bizzarri C. Minimal incidence of neonatal/infancy onset diabetes in Italy is 1:90,000 live births. Acta Diabetol. (2012) 49:405–8. 10.1007/s00592-011-0331-8 21953423 PMC3464369

[24] Aguilar-Bryan L Bryan J. Neonatal diabetes mellitus. Endocr Rev. (2008) 29:265–91. 10.1210/er.2007-0029 18436707 PMC2528857

[25] Gloyn AL Pearson ER Antcliff JF Proks P Bruining GJ Slingerland AS. Activating mutations in the gene encoding the ATP-sensitive potassium-channel subunit Kir6.2 and permanent neonatal diabetes. N Engl J Med. (2004) 350:1838–49. 10.1056/NEJMoa032922 15115830

[26] Habeb AM Flanagan SE Deeb A Al-Alwan I Alawneh H Balafrej AA. Permanent neonatal diabetes: different aetiology in Arabs compared to Europeans. Arch Dis Childhood. (2012) 97:721–3. 10.1136/archdischild-2012-301744 22859427

[27] Naylor RN Greeley SA Bell GI Philipson LH. Genetics and pathophysiology of neonatal diabetes mellitus. J Diabetes Investig. (2011) 2:158–69. 10.1111/j.2040-1124.2011.00106.x 24843477 PMC4014912

[28] Newsholme P Gaudel C Krause M. Mitochondria and diabetes. An intriguing pathogenetic role. Adv Exp Med Biol. (2012) 942:235–47. 10.1007/978-94-007-2869-1_10 22399425

[29] Gerbitz KD Gempel K Brdiczka D. Mitochondria and diabetes. Genetic, biochemical, and clinical implications of the cellular energy circuit. Diabetes. (1996) 45:113–26. 10.2337/diabetes.45.2.113 8549853

[30] Inoue H Tanizawa Y Wasson J Behn P Kalidas K Bernal-Mizrachi E. A gene encoding a transmembrane protein is mutated in patients with diabetes mellitus and optic atrophy (Wolfram syndrome). Nat Genet. (1998) 20:143–8. 10.1038/2441 9771706

[31] Delepine M Nicolino M Barrett T Golamaully M Lathrop GM Julier C. EIF2AK3, encoding translation initiation factor 2-alpha kinase 3, is mutated in patients with Wolcott-Rallison syndrome. Nat Genet. (2000) 25:406–9. 10.1038/78085 10932183

[32] Collin GB Marshall JD Ikeda A So WV Russell-Eggitt I Maffei P. Mutations in ALMS1 cause obesity, type 2 diabetes and neurosensory degeneration in Alstrom syndrome. Nat Genet. (2002) 31:74–8. 10.1038/ng867 11941369

[33] Oishi K Diaz GA Thiamine-responsive megaloblastic anemia syndrome. In: Adam MP Ardinger HH Pagon RA Wallace SE Bean LJH Stephens K, editors. GeneReviews^®^ [Internet]. Seattle, WA (1993). 20301459

[34] Green JS Parfrey PS Harnett JD Farid NR Cramer BC Johnson G. The cardinal manifestations of Bardet-Biedl syndrome, a form of Laurence-Moon-Biedl syndrome. N Engl J Med. (1989) 321:1002–9. 10.1056/NEJM198910123211503 2779627

[35] Cheng MH Anderson MS. Insights into type 1 diabetes from the autoimmune polyendocrine syndromes. Curr Opin Endocrinol Diabetes Obes. (2013) 20:271–8. 10.1097/MED.0b013e32836313eb 23770732 PMC4165040

[36] Finnish-German AC An autoimmune disease, APECED, caused by mutations in a novel gene featuring two PHD-type zinc-finger domains. Nat Genet. (1997) 17:399–403. 10.1038/ng1297-399 9398840

[37] Bennett CL Christie J Ramsdell F Brunkow ME Ferguson PJ Whitesell L. The immune dysregulation, polyendocrinopathy, enteropathy, X-linked syndrome (IPEX) is caused by mutations of FOXP3. Nat Genet. (2001) 27:20–1. 10.1038/83713 11137993

[38] Turkmen K Karagoz A Kucuk A. Sirtuins as novel players in the pathogenesis of diabetes mellitus. World J Diabetes. (2014) 5:894–900. 10.4239/wjd.v5.i6.894 25512793 PMC4265877

[39] Velayos T Martinez R Alonso M Garcia-Etxebarria K Aguayo A Camarero C. An activating mutation in STAT3 results in neonatal diabetes through reduced insulin synthesis. Diabetes. (2017) 66:1022–9. 10.2337/db16-0867 28073828

[40] Aldukhayel A. Prevalence of diabetic nephropathy among Type 2 diabetic patients in some of the Arab countries. Int J Health Sci. (2017) 11:1–4. 28293155 PMC5327670

[41] Zabetian A Kelli HM Echouffo-Tcheugui JB Narayan KM Ali MK. Diabetes in the Middle East and North Africa. Diabetes Res Clin Pract. (2013) 101:106–22. 10.1016/j.diabres.2013.03.010 23642969

[42] Farag YM Al Wakeel JS. Diabetic nephropathy in the Arab Gulf countries. Nephron Clin Pract. (2011) 119:c317–22; discussion: c22–3. 10.1159/000328909 22123454

[43] Al Dawish MA Robert AA Braham R Al Hayek AA Al Saeed A Ahmed RA. Diabetes mellitus in Saudi Arabia: a review of the recent literature. Curr Diabetes Rev. (2016) 12:359–68. 10.2174/1573399811666150724095130 26206092

[44] Zayed H Ouhtit A El Bekay R. An Arab registry for type 1 diabetes: global benefits for type 1 diabetes patients. Curr Med Res Opin. (2016) 32:1–4. 10.1080/03007995.2016.1198756 27264271

[45] Alotaibi A Perry L Gholizadeh L Al-Ganmi A. Incidence and prevalence rates of diabetes mellitus in Saudi Arabia: an overview. J Epidemiol Glob Health. (2017) 7:211–8. 10.1016/j.jegh.2017.10.001 29110860 PMC7384574

[46] Robert AA Al-Dawish A Mujammami M Dawish MAA. Type 1 diabetes mellitus in Saudi Arabia: a soaring epidemic. Int J Pediatr. (2018) 2018:9408370. 10.1155/2018/9408370 29853923 PMC5964576

[47] Alyafei F Soliman A Alkhalaf F Sabt A De Sanctis V Waseef R. Incidence of type 1 and type 2 diabetes, between 2012–2016, among children and adolescents in Qatar. Acta Bio Med Atenei Parmensis. (2018) 89:7–10. 10.23750/abm.v89iS4.7360 30049926 PMC6179092

[48] Usher-Smith JA Thompson M Ercole A Walter FM. Variation between countries in the frequency of diabetic ketoacidosis at first presentation of type 1 diabetes in children: a systematic review. Diabetologia. (2012) 55:2878–94. 10.1007/s00125-012-2690-2 22933123 PMC3464389

[49] Aminzadeh M Navidi N Valavi E Aletayeb SMH. Childhood onset type 1 diabetes at a tertiary hospital in south-western Iran during 2000-2015: Rapid increase in admissions and high prevalence of DKA at diagnosis. Primary Care Diabetes. (2019) 13:43–8. 10.1016/j.pcd.2018.07.013 30145190

[50] Vaxillaire M D P Bonnefond A Froguel P. Breakthroughs in monogenic diabetes genetics: from pediatric forms to young adulthood diabetes. Pediatric Endocrinol Rev. (2009) 6:405–17. 19396026

[51] Grosse J Hornstein H Manuwald U Kugler J Glauche I Rothe U Incidence of diabetic ketoacidosis of new-onset type 1 diabetes in children and adolescents in different countries correlates with human development index (HDI): an updated systematic review, meta-analysis, and meta-regression. Horm Metab Res. (2018) 50:209–22. 10.1055/s-0044-102090 29523007

[52] Tsertsvadze A Maglione M Chou R Garritty C Coleman C Lux L. Updating comparative effectiveness reviews: current efforts in AHRQ.s Effective Health Care Program. J Clin Epidemiol. (2011) 64:1208–15. 10.1016/j.jclinepi.2011.03.011 21684114

[53] Skelly A Hashimoto R Al-Khatib S Sanders-Schmidler G Fu R Brodt E AHRQ Technology Assessments. Catheter Ablation for Treatment of Atrial Fibrillation. Rockville, MD: Agency for Healthcare Research and Quality (US) (2015). 26225408

[54] Moher D Liberati A Tetzlaff J Altman DG The PG Preferred Reporting Items for Systematic Reviews and Meta-Analyses: The PRISMA Statement. PLOS Med. (2009) 6:e1000097 10.1371/journal.pmed.1000097 19621072 PMC2707599

[55] Tuomilehto J. The emerging global epidemic of type 1 diabetes. Curr Diabetes Rep. (2013) 13:795–804. 10.1007/s11892-013-0433-5 24072479

[56] Abduljabbar MA Aljubeh JM Amalraj A Cherian MP Incidence trends of childhood type 1 diabetes in eastern Saudi Arabia. Saudi Med J. (2010) 31:413–8. 20383419

[57] Habeb AM Al-Magamsi MS Halabi S Eid IM Shalaby S Bakoush O. High incidence of childhood type 1 diabetes in Al-Madinah, North West Saudi Arabia (2004–2009). Pediatr Diabetes. (2011) 12:676–81. 10.1111/j.1399-5448.2011.00765.x 21418457

[58] Al-Ghamdi AH Fureeh AA. Prevalence and clinical presentation at the onset of type 1 diabetes mellitus among children and adolescents in AL-Baha region, Saudi Arabia. J Pediatric Endocrinol Metab. (2018) 31:269–73. 10.1515/jpem-2017-0059 29537213

[59] Cherian MP Al-Kanani KA Al Qahtani SS Yesurathinam H Mathew AA Thomas VS. The rising incidence of type 1 diabetes mellitus and the role of environmental factors–three decade experience in a primary care health center in Saudi Arabia. J Pediatric Endocrinol Metab. (2010) 23:685–95. 10.1515/JPEM.2010.23.7.685 20857841

[60] Al-Herbish AS El-Mouzan MI Al-Salloum AA Al-Qurachi MM Al-Omar AA. Prevalence of type 1 diabetes mellitus in Saudi Arabian children and adolescents. Saudi Med J. (2008) 29:1285–8. 10.4103/0256-4947.51812 18813413

[61] Al-Rubeaan K. National surveillance for type 1, type 2 diabetes and prediabetes among children and adolescents: a population-based study (SAUDI-DM). J Epidemiol Commun Health. (2015) 69:1045–51. 10.1136/jech-2015-205710 26085648 PMC4680138

[62] Shaltout AA Wake D Thanaraj TA Omar DM Al-AbdulRazzaq D Channanath A. Incidence of type 1 diabetes has doubled in Kuwaiti children 0–14 years over the last 20 years. Pediatric diabetes. (2017) 18:761–6. 10.1111/pedi.12480 27981709

[63] Moussa MA Alsaeid M Refai TM Abdella N Al-Sheikh N Gomez JE. Factors associated with type 1 diabetes in Kuwaiti children. Acta Diabetol. (2005) 42:129–37. 10.1007/s00592-005-0192-0 16258736

[64] Alyafei F Soliman A Alkhalaf F Sabt A De Sanctis V Elsayed N. Clinical and biochemical characteristics of familial type 1 diabetes mellitus (FT1DM) compared to non-familial type 1 DM (NFT1DM). Acta Bio Med Atenei Parmensis. (2018) 89:27–31. 10.23750/abm.v89iS4.7358 30049929 PMC6179093

[65] Damanhouri LH Dromey JA Christie MR Nasrat HA Ardawi MS Robins RA. Autoantibodies to GAD and IA-2 in Saudi Arabian diabetic patients. Diabetic Med J Br Diabetic Assoc. (2005) 22:448–52. 10.1111/j.1464-5491.2005.01438.x 15787671

[66] Alyafei F Soliman A Alkhalaf F Sabt A De Sanctis V Elsayed N. Prevalence of beta-cell antibodies and associated autoimmune diseases in children and adolescents with type 1 diabetes (T1DM) versus type 2 diabetes (T2DM) in Qatar. Acta Biomed. (2018) 89:32–9. 10.23750/abm.v89iS4.7359 30049930 PMC6179090

[67] Al-Jenaidi FA Wakim-Ghorayeb SF Al-Abbasi A Arekat MR Irani-Hakime N Najm P. Contribution of selective HLA-DRB1/DQB1 alleles and haplotypes to the genetic susceptibility of type 1 diabetes among Lebanese and Bahraini Arabs. J Clin Endocrinol Metab. (2005) 90:5104–9. 10.1210/jc.2005-1166 15985473

[68] Saruhan-Direskeneli G Uyar FA Bas F Gunoz H Bundak R Saka N. HLA-DR and -DQ associations with insulin-dependent diabetes mellitus in a population of Turkey. Hum Immunol. (2000) 61:296–302. 10.1016/S0198-8859(99)00182-2 10689119

[69] Ercilla MG Guardia A Suarez B Arias MT Fabregat V Costa M. Identification of a new HLA-DRB1 allele (DRB1^*^0318) in three members of a Caucasian Spanish family. Tissue Antigens. (2001) 57:489–91. 10.1034/j.1399-0039.2001.057005489.x 11556978

[70] Gillespie KM Gale EA Bingley PJ. High familial risk and genetic susceptibility in early onset childhood diabetes. Diabetes. (2002) 51:210–4. 10.2337/diabetes.51.1.210 11756343

[71] Al-Harbi EM Abbassi AJ Tamim H al-Jenaidi F Kooheji M Kamal M. Specific HLA-DRB and -DQB alleles and haplotypes confer disease susceptibility or resistance in Bahraini type 1 diabetes patients. Clin Diagnostic Lab Immunol. (2004) 11:292–6. 10.1128/CDLI.11.2.292-296.2004 15013978 PMC371219

[72] Al-Hayek AA Robert AA Braham RB Turki AS Al-Sabaan FS. Frequency and associated risk factors of recurrent diabetic ketoacidosis among Saudi adolescents with type 1 diabetes mellitus. Saudi Med J. (2015) 36:216–20. 10.15537/smj.2015.2.10560 25719588 PMC4375701

[73] Shaltout AA Channanath AM Thanaraj TA Omar D Abdulrasoul M Zanaty N. Ketoacidosis at first presentation of type 1 diabetes mellitus among children: a study from Kuwait. Sci Rep. (2016) 6:27519. 10.1038/srep27519 27328757 PMC4916451

[74] Satti SA Saadeldin IY Dammas AS. Diabetic Ketoacidosis in children admitted to Pediatric Intensive Care Unit of King Fahad Hospital, Al-Baha, Saudi Arabia: Precipitating factors, epidemiological parameters and clinical presentation. Sudanese J Paediatrics. (2013) 13:24–30. Available online at: https://kopernio.com/viewer?doi=27493370&token=WzEzMDM3NjEsIjI3NDkzMzcwIl0.HVMv22LyWzf1_m2fP2QfnhILzz4 27493370 PMC4949937

[75] Naeem MA Al-Alem HA Al-Dubayee MS Al-Juraibah FN Omair A Al-Ruwaili AS. Characteristics of pediatric diabetic ketoacidosis patients in Saudi Arabia. Saudi Med J. (2015) 36:20–5. 10.15537/smj.2015.1.9763 25630000 PMC4362198

[76] Habib HS. Frequency and clinical characteristics of ketoacidosis at onset of childhood type 1 diabetes mellitus in Northwest Saudi Arabia. Saudi Med J. (2005) 26:1936–9. Available online at: https://www.researchgate.net/publication/7390173_Frequency_and_clinical_characteristics_of_ketoacidosis_at_onset_of_childhood_diabetes_mellitus_in_Northwest_Saudi_Arabia 16380776

[77] Abdul-Rasoul M Al-Mahdi M Al-Qattan H Al-Tarkait N Alkhouly M Al-Safi R. Ketoacidosis at presentation of type 1 diabetes in children in Kuwait: frequency and clinical characteristics. Pediatric diabetes. (2010) 11:351–6. 10.1111/j.1399-5448.2009.00600.x 19821943

[78] Kulaylat NA Narchi H. Clinical picture of childhood type 1 diabetes mellitus in the Eastern Province of Saudi Arabia. Pediatric Diabetes. (2001) 2:43–7. 10.1046/j.1399-543x.2001.020108.x 15016210

[79] Sayed MH Hegazi MA Abdulwahed K Moussa K El-Deek BS Gabel H. Risk factors and predictors of uncontrolled hyperglycemia and diabetic ketoacidosis in children and adolescents with type 1 diabetes mellitus in Jeddah, western Saudi Arabia. J Diabetes. (2017) 9:190–9. 10.1111/1753-0407.12404 27043144

[80] Al-Agha A Ocheltree A Shata N. Prevalence of hyperinsulinism, type 2 diabetes mellitus and metabolic syndrome among Saudi overweight and obese pediatric patients. Minerva Pediatr. (2012) 64:623–31. 23108324

[81] Punnose J Agarwal MM Bin-Uthman S. Type 2 diabetes mellitus among children and adolescents in Al-Ain: a case series. East Mediterr Health J. (2005) 11:788–97. Available online at: https://www.researchgate.net/publication/7081883_Type_2_diabetes_mellitus_among_children_and_adolescents_in_Al-Ain_A_case_series 16700395

[82] Moussa MA Alsaeid M Abdella N Refai TM Al-Sheikh N Gomez JE. Prevalence of type 2 diabetes mellitus among Kuwaiti children and adolescents. Med Princ Pract. (2008) 17:270–5. 10.1159/000129604 18523392

[83] Stancakova A Laakso M. Genetics of Type 2 diabetes. Endocr Dev. (2016) 31:203–20. 10.1159/000439418 26824439

[84] O'Beirne SL Salit J Rodriguez-Flores JL Staudt MR Abi Khalil C Fakhro KA Type 2 diabetes risk Allele Loci in the Qatari population. PLoS ONE. (2016) 11:e0156834 10.1371/journal.pone.0156834 27383215 PMC4934876

[85] Habeb AM Al-Magamsi MS Eid IM Ali MI Hattersley AT Hussain K. Incidence, genetics, and clinical phenotype of permanent neonatal diabetes mellitus in northwest Saudi Arabia. Pediatric Diabetes. (2012) 13:499–505. 10.1111/j.1399-5448.2011.00828.x 22060631

[86] Deeb A Habeb A Kaplan W Attia S Hadi S Osman A. Genetic characteristics, clinical spectrum, and incidence of neonatal diabetes in the Emirate of AbuDhabi, United Arab Emirates. Am J Med Genet Part A. (2016) 170:602–9. 10.1002/ajmg.a.37419 26463504

[87] Abbasi F Habibi M Enayati S Bitarafan F Razzaghy-Azar M Sotodeh A. A genotype-first approach for clinical and genetic evaluation of Wolcott-Rallison syndrome in a large cohort of iranian children with neonatal diabetes. Can J Diabetes. (2018) 42:272–5. 10.1016/j.jcjd.2017.06.009 28843469

[88] Al Senani A Hamza N Al Azkawi H Al Kharusi M Al Sukaiti N Al Badi M. Genetic mutations associated with neonatal diabetes mellitus in Omani patients. J Pediatric Endocrinol Metab. (2018) 31:195–204. 10.1515/jpem-2017-0284 29329106 PMC6853791

[89] Elkholy S Lardhi AA Do we need to test for maturity onset diabetes of the young among newly diagnosed diabetics in Saudi Arabia? Int J Diabetes Mellitus. (2015) 3:51–6. 10.1016/j.ijdm.2011.01.006

[90] Charbonnier LM Janssen E Chou J Ohsumi TK Keles S Hsu JT. Regulatory T-cell deficiency and immune dysregulation, polyendocrinopathy, enteropathy, X-linked-like disorder caused by loss-of-function mutations in LRBA. J Allergy Clin Immunol. (2015) 135:217–27. 10.1016/j.jaci.2014.10.019 25468195 PMC4289093

[91] Hussain T Akle M Nagelkerke N Deeb A. Comparative study on treatment satisfaction and health perception in children and adolescents with type 1 diabetes mellitus on multiple daily injection of insulin, insulin pump and sensor-augmented pump therapy. SAGE Open Med. (2017) 5:2050312117694938. 10.1177/2050312117694938 28321303 PMC5347412

[92] Asma D Samar A-A Salima A Mohamed E-A Jamal A-J Hana Y Important determinants of diabetes control in insulin pump therapy in patients with type 1 diabetes mellitus. Diabetes Technol Therap. (2015) 17:166–70. 10.1089/dia.2014.0224 25513744

[93] Al-Agha AE Kafi SE Zain Aldeen AM Khadwardi RH. Flash glucose monitoring system may benefit children and adolescents with type 1 diabetes during fasting at Ramadan. Saudi Med J. (2017) 38:366–71. 10.15537/smj.2017.4.18750 28397942 PMC5447188

[94] Alamoudi R Alsubaiee M Alqarni A Saleh Y Aljaser S Salam A. Comparison of insulin pump therapy and multiple daily injections insulin regimen in patients with type 1 diabetes during ramadan fasting. Diabetes Technol Ther. (2017) 19:349–54. 10.1089/dia.2016.0418 28296467

[95] Petrovski G Al Khalaf F Hussain K Campbell J El Awwa A. Continuous subcutaneous insulin infusion characteristics in type 1 diabetes children and adolescents in Qatar. Diabetes Ther. (2018) 9:2091–8. 10.1007/s13300-018-0510-5 30220038 PMC6167275

[96] Petrovski G Al Khalaf F Hussain K Campbell J. Optimizing a hybrid closed loop system in type 1 diabetes: a case report. Diabetes Ther. (2018) 9:2173–7. 10.1007/s13300-018-0473-6 30030688 PMC6167286

[97] Seyyed MT Farkhondeh A Hamid M Seyyed RK Mahtab N A study of population based diabetes registry in developed countries. JOJ Nurs Health Care. (2018) 8:555732 10.19080/JOJNHC.2018.08.555732

[98] Rankin J Best K Disease registers in England. Paediatr Child Health. (2014) 24:337–42. 10.1016/j.paed.2014.02.002

[99] Park BZ Cantrell L Hunt H Farris RP Schumacher P Bauer UE. State public health actions to prevent and control diabetes, heart disease, obesity and associated risk factors, and promote school health. Prevent Chronic Dis. (2017) 14:E127. 10.5888/pcd14.160437 29215978 PMC5724997

[100] Gliklich R Dreyer N Leavy M Registries for Evaluating Patient Outcomes: A User's Guide. 3rd ed Rockville, MD: Agency for Healthcare Research and Quality (US) (2014). Available online at: https://effectivehealthcare.ahrq.gov/sites/default/files/registries-guide-3rd-edition-vol-2-140430.pdf 24945055

[101] Bartlett C Holenstein F Sterne J Egger M Jüni P. Direction and impact of language bias in meta-analyses of controlled trials: empirical study. Int J Epidemiol. (2002) 31:115–23. 10.1093/ije/31.1.115 11914306

[102] Egger M Zellweger-Zähner T Schneider M Junker C Lengeler C Antes G. Language bias in randomised controlled trials published in English and German. Lancet. (1997) 350:326–9. 10.1016/S0140-6736(97)02419-7 9251637

