# Supplementary material for: A Systematic Review of Childhood Diabetes Research in the Middle East Region
Source: Front Endocrinol (Lausanne). 2019 Nov 19;10:805. doi: 10.3389/fendo.2019.00805 (PMC6882272; doi:10.3389/fendo.2019.00805)
Supplement: Supplementary file 1 [file Data_Sheet_1.pdf]

A Systematic Review of Childhood Diabetes Research in the Middle East Region

Saras Saraswathi[1#], Sara Al-Khawaga[1, 3#], Naser Elkum[2] and Khalid Hussain[1*]
[1]Department of Pediatrics, Division of Endocrinology, Sidra Medicine, Doha, Qatar
[2] Clinical Research Center, Biostatistics Sec, Research Services, Sidra Medicine, Doha, Qatar
[3] College of Health & Life Sciences, Hamad Bin Khalifa University, Qatar Foundation, Education City, Doha, Qatar

Appendix A – Evidence Tables for Review papers used to fill in gaps in knowledge and include these in our review.

(Table on the next page)

Key:

COI = Conflict of Interest
DKA = Diabetic ketoacidosis
DM = Diabetes Mellitus
KSA = Kingdom of Saudi Arabia
NG = Not Given
NA = Not Applicable
T1DM = Type 1 Diabetes Mellitus
T2DM = Type 2 Diabetes Mellitus

The table gives the reviews that were used for content comparison and to fill in the gaps in knowledge for our own review. Although these studies are ranked, they were not included in the overall qualitative analysis. Only those studies pertaining to DM (not reviews) were included in the meta-analysis.

#	Author (year)	Study Design	Study Characteristics	Patient Characteristics	Quality	Study Standard	Study Quality
		**Type of Study (Regional/Country-wide) [Multi-Center]**	**Study Period (Length of Study)**	**No. of Participants (Age range) [Male/Female] {Health Burden}**	**Predefined outcomes (confounding / bias) [Types of DM]**	**Ethics Approval (COI) [Funding]**	**Quality scale based on our review needs**
1	Robert, A.A et.al. 2017	Review paper (country - wide) [yes]	review of mulitple studies (long)	emphasis on KSA	Yes (NG) [Only T1DM and T2DM]	(NG)	level-2
2	Aldukhayel, A 2017	Review paper (country - wide) [yes]	review of mulitple studies (long)	(NG) [NG]	Yes (only four Arab countires) [Only T2DM]	(NG)	level-2
3	Al Dawish MA et.al. 2016	Review paper (country - wide) [yes]	review of mulitple studies (long)	(NG) [NG]	Yes (Saudi Arabia) [Only DM]	(NG)	level-2
4	Zayed H et.al. 2016	Review paper (Many countries) [yes]	NA	> 10000 (NG) [NG]	Yes (22 Countries) [Only T1DM]	NG	level-2
5	Zabetian, A et.al. 2014	Review paper (Many countries) [yes]	NA	> 1000 (0-14 yrs) {13.64 billion USD}	Yes (MENA regions) [Only T2DM]	Yes (No) [Yes]	level-2

6	Farag, Y.M<br>2011	Review paper<br>(Many countries)<br>[yes]	NA	> 1000<br>Adults<br>Significant cost	Yes<br>(Adults only)<br>[Only T1DM and T2DM]	unknown	level-4
7	Alotaibi, A., et al<br>2017	Descriptive Review paper<br>(country - wide)<br>[yes]	25 years	> 1000<br>(0 to 14 yrs.)<br>[Yes]<br>0.87 billion USD	Yes<br>(Saudi Arabia)<br>[Only T1DM and T2DM]	unknown	level-1
8	Robert, A.A., et al.,<br>2018	Review of literature	25 years	NA	Yes<br>(Saudi Arabia)<br>[Only T1DM and T2DM]	unknown	level-1
9	Usher-Smith, J.A. et. al.<br>2012	Review of literature	NA	65 studies<br>65000 patients	Yes<br>(Saudi Arabia)<br>[Only T1DM and DKA]	Yes<br>(No)<br>[Yes]	level-1
