# Supplementary material for: A Systematic Review of Childhood Diabetes Research in the Middle East Region
Source: Front Endocrinol (Lausanne). 2019 Nov 19;10:805. doi: 10.3389/fendo.2019.00805 (PMC6882272; doi:10.3389/fendo.2019.00805)
Supplement: Supplementary file 2 [file Data_Sheet_2.PDF]

A Systematic Review of Childhood Diabetes Research in the Middle East Region

Saras Saraswathi[1#], Sara Al-Khawaga[1, 3#], Naser Elkum[2] and Khalid Hussain[1*]
[1]Department of Pediatrics, Division of Endocrinology, Sidra Medicine, Doha, Qatar
[2] Clinical Research Center, Biostatistics Sec, Research Services, Sidra Medicine, Doha, Qatar
[3] College of Health & Life Sciences, Hamad Bin Khalifa University, Qatar Foundation, Education City, Doha, Qatar

APPENDIX B

List of studies in the Middle East Region that were included in our review

1. Robert, A.A., et al., *Type 2 Diabetes Mellitus in Saudi Arabia: Major Challenges and Possible Solutions.* Curr Diabetes Rev, 2017. **13**(1): p. 59-64.
2. Habeb, A.M., et al., *Permanent neonatal diabetes: different aetiology in Arabs compared to Europeans.* Arch Dis Child, 2012. **97**(8): p. 721-3.
3. Alyafei, F., et al., *Incidence of type 1 and type 2 diabetes, between 2012-2016, among children and adolescents in Qatar.* Acta Biomed, 2018. **89**(S5): p. 7-10.
4. Aldukhayel, A., *Prevalence of diabetic nephropathy among Type 2 diabetic patients in some of the Arab countries.* Int J Health Sci (Qassim), 2017. **11**(1): p. 1-4.
5. Al Dawish, M.A., et al., *Diabetes Mellitus in Saudi Arabia: A Review of the Recent Literature.* Curr Diabetes Rev, 2016. **12**(4): p. 359-368.
6. Aminzadeh, M., et al., *Childhood onset type 1 diabetes at a tertiary hospital in south-western Iran during 2000-2015: Rapid increase in admissions and high prevalence of DKA at diagnosis.* Prim Care Diabetes, 2018.
7. Zayed, H., A. Ouhtit, and R. El Bekay, *An Arab registry for type 1 diabetes: global benefits for type 1 diabetes patients.* Curr Med Res Opin, 2016: p. 1-4.
8. Zabetian, A., et al., *Diabetes in the Middle East and North Africa.* Diabetes Res Clin Pract, 2013. **101**(2): p. 106-22.
9. Farag, Y.M. and J.S. Al Wakeel, *Diabetic nephropathy in the Arab Gulf countries.* Nephron Clin Pract, 2011. **119**(4): p. c317-22; discussion c322-3.
10. Alotaibi, A., et al., *Incidence and prevalence rates of diabetes mellitus in Saudi Arabia: An overview.* J Epidemiol Glob Health, 2017. **7**(4): p. 211-218.
11. Al-Ghamdi, A.H. and A.A. Fureeh, *Prevalence and clinical presentation at the onset of type 1 diabetes mellitus among children and adolescents in AL-Baha region, Saudi Arabia.* J Pediatr Endocrinol Metab, 2018. **31**(3): p. 269-273.
12. Al-Herbish, A.S., et al., *Prevalence of type 1 diabetes mellitus in Saudi Arabian children and adolescents.* Saudi Med J, 2008. **29**(9): p. 1285-8.
13. Shaltout, A.A., et al., *Incidence of type 1 diabetes has doubled in Kuwaiti children 0-14 years over the last 20 years.* Pediatr Diabetes, 2017. **18**(8): p. 761-766.
14. Damanhouri, L.H., et al., *Autoantibodies to GAD and IA-2 in Saudi Arabian diabetic patients.* Diabet Med, 2005. **22**(4): p. 448-52.

15. Alyafei, F., et al., *Prevalence of beta-cell antibodies and associated autoimmune diseases in children and adolescents with type 1 diabetes (T1DM) versus type 2 diabetes (T2DM) in Qatar.* Acta Biomed, 2018. **89**(S5): p. 32-39.
16. Al-Jenaidi, F.A., et al., *Contribution of selective HLA-DRB1/DQB1 alleles and haplotypes to the genetic susceptibility of type 1 diabetes among Lebanese and Bahraini Arabs.* J Clin Endocrinol Metab, 2005. **90**(9): p. 5104-9.
17. Lila Ahmed Albishi, M.M.A., Sawsan Mohammed Albelwi, Rihab Hafez Osman, Nehal Abdulgadir Ahmed, Mohammed Fararjeh, *Clinical Demographic Patterns of Type 1 Diabetes in Saudi Children in Tabuk City, 2000-2010* Journal of Diabetes Mellitus, 2017. **41-54**(7).
18. Al-Agha, A., A. Ocheltree, and N. Shata, *Prevalence of hyperinsulinism, type 2 diabetes mellitus and metabolic syndrome among Saudi overweight and obese pediatric patients.* Minerva Pediatr, 2012. **64**(6): p. 623-31.
19. Punnose, J., et al., *Childhood and adolescent diabetes mellitus in Arabs residing in the United Arab Emirates.* Diabetes Res Clin Pract, 2002. **55**(1): p. 29-33.
20. Punnose, J., M.M. Agarwal, and S. Bin-Uthman, *Type 2 diabetes mellitus among children and adolescents in Al-Ain: a case series.* East Mediterr Health J, 2005. **11**(4): p. 788-97.
21. Moussa, M.A., et al., *Prevalence of type 2 diabetes mellitus among Kuwaiti children and adolescents.* Med Princ Pract, 2008. **17**(4): p. 270-5.
22. Ali, B.A., et al., *The Frequency of Type 2 Diabetes Mellitus among Diabetic Children in El Minia Governorate, Egypt.* Sultan Qaboos Univ Med J, 2013. **13**(3): p. 399-403.
23. Habeb, A.M., et al., *Incidence, genetics, and clinical phenotype of permanent neonatal diabetes mellitus in northwest Saudi Arabia.* Pediatr Diabetes, 2012. **13**(6): p. 499-505.
24. Deeb, A., et al., *Genetic characteristics, clinical spectrum, and incidence of neonatal diabetes in the Emirate of AbuDhabi, United Arab Emirates.* Am J Med Genet A, 2016. **170**(3): p. 602-9.
25. Abbasi, F., et al., *A Genotype-First Approach for Clinical and Genetic Evaluation of Wolcott-Rallison Syndrome in a Large Cohort of Iranian Children With Neonatal Diabetes.* Can J Diabetes, 2018. **42**(3): p. 272-275.
26. Al Senani, A., et al., *Genetic mutations associated with neonatal diabetes mellitus in Omani patients.* J Pediatr Endocrinol Metab, 2018. **31**(2): p. 195-204.
27. Elkholy, S. and A.A. Lardhi, *Do we need to test for maturity onset diabetes of the young among newly diagnosed diabetics in Saudi Arabia?* International Journal of Diabetes Mellitus, 2015. **3**(1): p. 51-56.
28. Hussain, T., et al., *Comparative study on treatment satisfaction and health perception in children and adolescents with type 1 diabetes mellitus on multiple daily injection of insulin, insulin pump and sensor-augmented pump therapy.* SAGE Open Med, 2017. **5**: p. 2050312117694938.
29. Asma, D., et al., *Important Determinants of Diabetes Control in Insulin Pump Therapy in Patients with Type 1 Diabetes Mellitus.* Diabetes Technology & Therapeutics, 2015. **17**(3): p. 166-170.
30. Al-Agha, A.E., et al., *Flash glucose monitoring system may benefit children and adolescents with type 1 diabetes during fasting at Ramadan.* Saudi Med J, 2017. **38**(4): p. 366-371.

31. Alamoudi, R., et al., *Comparison of Insulin Pump Therapy and Multiple Daily Injections Insulin Regimen in Patients with Type 1 Diabetes During Ramadan Fasting.* Diabetes Technol Ther, 2017. **19**(6): p. 349-354.
32. Petrovski, G., et al., *Continuous Subcutaneous Insulin Infusion Characteristics in Type 1 Diabetes Children and Adolescents in Qatar.* Diabetes Ther, 2018. **9**(5): p. 2091-2098.
33. Petrovski, G., et al., *Optimizing a Hybrid Closed Loop System in Type 1 Diabetes: A Case Report.* Diabetes Ther, 2018. **9**(5): p. 2173-2177.
34. Robert, A.A., et al., *Type 1 Diabetes Mellitus in Saudi Arabia: A Soaring Epidemic.* Int J Pediatr, 2018. **2018**: p. 9408370.
35. Cherian, M.P., et al., *The rising incidence of type 1 diabetes mellitus and the role of environmental factors--three decade experience in a primary care health center in Saudi Arabia.* J Pediatr Endocrinol Metab, 2010. **23**(7): p. 685-95.
36. Abduljabbar, M.A., et al., *Incidence trends of childhood type 1 diabetes in eastern Saudi Arabia.* Saudi Med J, 2010. **31**(4): p. 413-8.
37. Habeb, A.M., et al., *High incidence of childhood type 1 diabetes in Al-Madinah, North West Saudi Arabia (2004-2009).* Pediatr Diabetes, 2011. **12**(8): p. 676-81.
38. Al-Rubeaan, K., *National surveillance for type 1, type 2 diabetes and prediabetes among children and adolescents: a population-based study (SAUDI-DM).* J Epidemiol Community Health, 2015. **69**(11): p. 1045-51.
39. Moussa, M.A., et al., *Factors associated with type 1 diabetes in Kuwaiti children.* Acta Diabetol, 2005. **42**(3): p. 129-37.
40. Alyafei, F., et al., *Clinical and biochemical characteristics of familial type 1 diabetes mellitus (FT1DM) compared to non-familial type 1 DM (NFT1DM).* Acta Biomed, 2018. **89**(S5): p. 27-31.
41. Saruhan-Direskeneli, G., et al., *HLA-DR and -DQ associations with insulin-dependent diabetes mellitus in a population of Turkey.* Hum Immunol, 2000. **61**(3): p. 296-302.
42. Al-Harbi, E.M., et al., *Specific HLA-DRB and -DQB alleles and haplotypes confer disease susceptibility or resistance in Bahraini type 1 diabetes patients.* Clin Diagn Lab Immunol, 2004. **11**(2): p. 292-6.
43. Usher-Smith, J.A., et al., *Variation between countries in the frequency of diabetic ketoacidosis at first presentation of type 1 diabetes in children: a systematic review.* Diabetologia, 2012. **55**(11): p. 2878-94.
44. Al-Hayek, A.A., et al., *Frequency and associated risk factors of recurrent diabetic ketoacidosis among Saudi adolescents with type 1 diabetes mellitus.* Saudi Med J, 2015. **36**(2): p. 216-20.
45. Shaltout, A.A., et al., *Ketoacidosis at first presentation of type 1 diabetes mellitus among children: a study from Kuwait.* Sci Rep, 2016. **6**: p. 27519.
46. Satti, S.A., I.Y. Saadeldin, and A.S. Dammas, *Diabetic Ketoacidosis in children admitted to Pediatric Intensive Care Unit of King Fahad Hospital, Al-Baha, Saudi Arabia: Precipitating factors, epidemiological parameters and clinical presentation.* Sudan J Paediatr, 2013. **13**(2): p. 24-30.
47. Naeem, M.A., et al., *Characteristics of pediatric diabetic ketoacidosis patients in Saudi Arabia.* Saudi Med J, 2015. **36**(1): p. 20-5.
48. Habib, H.S., *Frequency and clinical characteristics of ketoacidosis at onset of childhood type 1 diabetes mellitus in Northwest Saudi Arabia.* Saudi Med J, 2005. **26**(12): p. 1936-9.

49. Abdul-Rasoul, M., et al., *Kestoacidosis at presentation of type 1 diabetes in children in Kuwait: frequency and clinical characteristic.* Pediatr Diabetes, 2010. **11**(5): p. 351-6.
50. Kulaylat, N.A. and H. Narchi, *Clinical picture of childhood type 1 diabetes mellitus in the Eastern Province of Saudi Arabia.* Pediatr Diabetes, 2001. **2**(1): p. 43-7.
51. Sayed, M.H., et al., *Risk factors and predictors of uncontrolled hyperglycemia and diabetic ketoacidosis in children and adolescents with type 1 diabetes mellitus in Jeddah, western Saudi Arabia.* J Diabetes, 2017. **9**(2): p. 190-199.
52. Stancakova, A. and M. Laakso, *Genetics of Type 2 Diabetes.* Endocr Dev, 2016. **31**: p. 203-20.
53. O'Beirne, S.L., et al., *Type 2 Diabetes Risk Allele Loci in the Qatari Population.* PLOS ONE, 2016. **11**(7): p. e0156834.
