# Supplementary material for: A Systematic Review of Childhood Diabetes Research in the Middle East Region
Source: Front Endocrinol (Lausanne). 2019 Nov 19;10:805. doi: 10.3389/fendo.2019.00805 (PMC6882272; doi:10.3389/fendo.2019.00805)
Supplement: Supplementary file 3 [file Data_Sheet_3.PDF]

A Systematic Review of Childhood Diabetes Research in the Middle East Region

Saras Saraswathi[1#], Sara Al-Khawaga[1, 3#], Naser Elkum[2] and Khalid Hussain[1*]
[1]Department of Pediatrics, Division of Endocrinology, Sidra Medicine, Doha, Qatar
[2] Clinical Research Center, Biostatistics Sec, Research Services, Sidra Medicine, Doha, Qatar
[3] College of Health & Life Sciences, Hamad Bin Khalifa University, Qatar Foundation, Education City, Doha, Qatar

APPENDIX C - Sample data collection form

Key Questions Addressed

Publications

1. Study design (randomized trial, prospective, retrospective, cross-sectional)
2. Funding source
3. Declaration of Conflict of interest by all authors
4. Region or Country
5. One center or multi-center study
6. Objective of the study
7. Inclusion and exclusion criteria used
8. Length of study
9. Period of study (2000 to 2018)
10. Types of Diabetes Mellitus were discussed: NDM, T1DM, T2DM, MODY, Maternally inherited Diabetes, Autoimmune monogenic DM.
11. Discussion of results

Details of cohort

1. Number of participants in the study
2. The population covered in this study.
3. Race/ethnic group(s) (% (n/N) - Any Arabic population?
4. Age (mean +/− SD)

5. Gender: Male: n/N (%); Female: n/N (%)
6. Any confounding factors

Quality of results:

1. Type of result - Qualitative or quantitative
2. Is the study report based on pre-specified outcomes?
3. Quality guideline used
4. Any bias discovered in the results, conduct of study, population mix?

Rating of Studies

1. Many types of diabetes discussed, no bias, global or country wide study, representative mix of patients, long study period, no conflict of interest (COI) – **level-1 quality study**
2. Slight bias, multi-center study, reasonable period, most of the population included, no COI – **level-2 quality study**
3. Slight bias, single center study, short period, some section of population included, no COI – **level-3 quality study**
4. Bias, single center, very short, not a good mix of population, COI – **level-4 quality study**

All studies were included in this review, since missing information was not a criteria used to eliminate studies. These rankings were used to determine the degree of contribution that each made to the article as a whole.

Reference:

Andrea Skelly, PhD, MPH, Robin Hashimoto, PhD, Sana Al-Khatib, MD, MHS, Gillian Sanders-Schmidler, PhD, Rochelle Fu, PhD, Erika Brodt, BS, and Marian McDonagh, PharmD. “Appendix D: Catheter Ablation for Treatment of Atrial Fibrillation: Technology Assessment Report, Agency for Healthcare Research and Quality (US); 2015
