# Supplementary material for: A Systematic Review of Childhood Diabetes Research in the Middle East Region
Source: Front Endocrinol (Lausanne). 2019 Nov 19;10:805. doi: 10.3389/fendo.2019.00805 (PMC6882272; doi:10.3389/fendo.2019.00805)
Supplement: Supplementary file 4 [file Data_Sheet_4.PDF]

A Systematic Review of Childhood Diabetes Research in the Middle East Region

Saras Saraswathi[1#], Sara Al-Khawaga[1, 3#], Naser Elkum[2] and Khalid Hussain[1*]
[1]Department of Pediatrics, Division of Endocrinology, Sidra Medicine, Doha, Qatar
[2] Clinical Research Center, Biostatistics Sec, Research Services, Sidra Medicine, Doha, Qatar
[3] College of Health & Life Sciences, Hamad Bin Khalifa University, Qatar Foundation, Education City, Doha, Qatar

Appendix D – Evidence Tables for studies used to fill in our review.

(Table on the next page)

Key:

COI = Conflict of Interest
DKA = Diabetic ketoacidosis
DM = Diabetes Mellitus
KSA = Kingdom of Saudi Arabia
NG = Not Given
NA = Not Applicable
T1DM = Type 1 Diabetes Mellitus
T2DM = Type 2 Diabetes Mellitus

The table gives the studies that were used in our analysis.

	Study	Study Design	Study Characteristics	Patient Characteristics	Study Quality	Study Standard	Quality Score
#	Author<br>(year)	Type of Study<br>(Regional/Country-wide)<br>[Multi-Center]	Study Period<br>(Length of Study)	No. of Participants<br>(Age range)<br>[Male/Female]<br>{Health Burden}	Predefined outcomes<br>(confounding/bias)<br>[Types of DM]	Ethics Approval<br>(Conflict of Interest)<br>[Funding]	Quality scale based on our review needs
1	Habeb et. al.<br>(2012)	Retrospective Observational, Molecular<br>(world-wide)<br>[yes]	5 years<br>(A point in time)	88 Arab / 77 British<br>(NG)<br>(NG)	Yes<br>(no gender)<br>[NDM]	Yes<br>(No)<br>[Yes]	level-1
2	Alyafei F. et.al.<br>(2018)	Prospective cohort study	4 years<br>(4 years)	(T1DM -0.5-14,T2DM -5 to 14 years)<br>[ ]	Yes<br>(no gender)<br>[T1DM (440), T2DM (45)]	NG<br>(NG)<br>[NG]	level-2
3	Aletayeb MA, et.al.<br>2018	Prospective cohort study<br>(local)<br>[No]	15 years<br>(long)	914<br>(age < 15)<br>(NG)	Yes<br><br>[Only DM and DKA]	NG<br>(NG)<br>[NG]	level-3
4	Al-Ghamdi A.H. et. al.<br>2018	Retrospective study<br>(local)<br>[No]	10 years<br>(long)	471<br>(0 to 19 years)<br>[Yes]<br>{NG}	Yes<br>(Saudi Arabia)<br>[OnlyT1DM]	NG<br>(No)<br>[No]	level-2
5	Al-Herbish, A.S., et al.,<br>2008	Retrospective study<br>(nationwide)<br>[Yes]	7 years<br>(long)	45682<br>(0 to 19 years)<br>[Yes]	Yes<br>(Saudi Arabia)<br>[OnlyT1DM]	Unknown	level-2

6	Shaltout, A.A., et al. 2018	Prospective study (nationwide) [Yes]	2years (medium period)	515 Kuwaiti children (247 boys and 268 girls) (0 to 14 years)	Yes (Kuwait) [OnlyT1DM]	Unknown	level-2
7	Damanhouri, L.H., et al., 2005	Prospective cohort study (local) [No]		99 (NG) [NG] {NG}	Yes (Jeddah) [Only auto antibodies in T1DM and T2DM]	Unknown	level-3
8	Alyafei, F., et al. 2018	Retrospective study	5 years (long)	T1DM (n=431) and T2DM (n=59) 0.5-16 years [no gender given]	Yes (Qatar) [T1DM and T2DM and antibodies]	Unknown	level-2
9	16. Al-Jenaidi, F.A., et al. 2005	Cross-sectional Retrospective study (two countries) (multiple centers)	Unknown	T1DM: 126/ Normal:126 Bahrain T1DM:78/ Normal: 111 Lebanese (NG) [NG]	Yes (Bahrain and Lebanon) [T1DM and HLA]	Unknown	level-3
10	Albishi, M.M.A., 2017	Retrospective Observational study (local) (One hospital)	10 years (long)	313 patients (< 12 years) [yes]	Yes (only Saudi Arabia) [predominantly girls] [OnlyT1DM]	NG (No) [No]	level-3
11	Al-Agha, A., A. 2012	Retrospective Cross-sectional study (single city) [one center]	5 years (long)	387 patients (2 to 18 years) [No gender]	Yes (only single center in Saudi Arabia) [OnlyT2DM]	Unknown	level-3

12	Punnose, J., et al. 2002	Retrospective study (single city) [one center]	9 years (long)	40 (0 to 18 years) [no gender]	Yes (Only UAE) [T1DM and T2DM]	Unknown	level-4
13	Punnose, J. et. al. 2005	Retrospective study (single city) [one center]	11 years (long)	96 (11 T2DM) (adolescents) [no gender]	Yes (no: mix of nationalities) [Only T2DM]	Unknown	level-4
14	Moussa, M.A., et al. 2008	Retrospective study (national) [multi center]	2 years (moderate)	128,918 (45 with T2DM) (6 to 18 yrs.) [age and gender given]	Yes (no; low p-values) [Only T2DM]	Unknown	level-2
15	Ali, B.A., et al. 2013	Prospective study (single center) [No]	1 year (fair)	T2DM is no longer a disease of adults but can also occur in children and adolescents.	Yes (only one center) [Only T2DM]	Unknown	level-3
16	Habeb AM et. al. 2012	Retrospective study Molecular basis for identifying genetic factors (national)	Over 10 years	17 patients (11 consanguineous families) (0-6 months) [no gender - NA]	Yes (no gender) [NDM]	Yes (No) [Yes]	level-1
17	Deeb, A., et al. 2016	Retrospective study Molecular basis for identifying genetic factors (national)	Over 28 years	25 patients (0-6 months) [no gender - NA]	Yes (no gender) [NDM]	Unknown	level-1

18	Abbasi, F., et al. 2018	Retrospective study Molecular basis for identifying genetic factors (national)	Unknown	60 (11 NDM) (0-6 months) [no gender - NA]	Yes (no gender) [NDM]	Unknown	level-1
19	Al Senani, A., et al. 2018	Retrospective study Molecular basis for identifying genetic factors (national)	8 years	24 patients (0-6 months) [no gender - NA]	Yes (no gender) [NDM]	Unknown	level-1
20	Elkholy, S. and A.A. Lardhi 2015	Literature Search	NA	NA	NA	Unknown	level-4
21	Hussain, T., et al. 2017	Comparative Study	NA	72 patients 6-15 years [no gender]	Yes (no gender) [DM]	Unknown	level-4
22	Asma, D., et al. 2015	Retrospective study, technology evaluation (local) [one center]	8-12-week	72 patients (50 children) [no gender]	Yes (no gender) [DM]	Unknown	level-4
23	Al-Agha, A.E., et al. 2017	Prospective, technology evaluation (local) (one center)	two months	51 patients (0-18) [no gender]	Yes (no gender) [T1DM]	Unknown	level-3
24	Alamoudi, R., et al. 2014	Prospective comparative study	two months	156 (61/95) (No age given) [No gender]	Yes (no gender) [T1DM]	Unknown	level-4
25	Petrovski, G., et al. 2018	Retrospective study, technology evaluation (local) [one center]	2 years (moderate)	138 (5-13 yrs.) [62/76 M/F]	Yes (small number of patients) [T1DM]	Yes (No) [Institutional]	level-1

26	Petrovski, G., et al. 2018	Retrospective study, technology evaluation (local) [one center]		one person 11 yrs.	Yes one person T1DM	Yes (No) [authors]	NA
27	Cherian, M.P., et al. 2010	Retrospective study (local) [One Center]	29 years (long)	119 patients (0-18) [not given]	Yes (one center) [T1DM]	Unknown	level-3
28	Abduljabbar, M.A., et al. 2010	Observational study (one region) [one center]	17 years (long)	438 patients (<15 years) [45:55%]	Yes (one center) [T1DM]	Unknown	level-3
29	Habeb, A.M., et al. 2011	Observational study (one region) [one center]	5 years (long)	419 patients (0-12 yrs.) [170/249]	Yes (one center, low p-values) [T1DM]	Unknown	level-1
30	Al-Rubeaan, K., 2015	Prospective Observational study (nationwide) [Yes]	2 years (moderate)	23 523 (< 18 years) [yes]	Yes (one center) [T1DM and T2DM]	Yes (no, low p-values for T1DM) [Yes]	level-1
31	Moussa, M.A., et al. 2005	Prospective Observational study (nationwide) [Yes]	NG	T1DM:348/348 Normal (6-18 yrs.) (131 males, 217 females)	Yes (one center) [T1DM and T2DM]	Unknown	level-3
32	Alyafei, F., et al. 2018	cross sectional descriptive study (national)	14 years (long)	T1DM 111: normal 431 (6-16 yrs.) [58.5: 41.5}	Yes (one center) [T1DM and T2DM]	Unknown	level-2
33	Saruhan-Direskeneli, G., et al. 2000	Molecular study	NA	IDDM: 178/ normal: 248	Yes (one center) [T1DM and HLA]	Unknown	level-1

34	Al-Harbi, E.M., et al. 2004	Molecular study	NA	T1DM - 107 : normal - 88 (mean ~ 15 yrs.) [not given]	Yes (one center) [T1DM and HLA]	Unknown	level-1
35	Al-Hayek, A.A., et al. 2015	A Cross-sectional study	1 yr. 4 months (moderate)	T1DM -103 (13-18 years) [57 males, 46 females]	Yes (one center) [T1DM and DKA]	Unknown	level-3
36	Shaltout, A.A., et al. 2016	Prospective study (nationwide) [Yes]	2 years (moderate)	679 (0-14 yrs.) [no gender association]	Yes (national) [T1DM and DKA]	yes (no) [Yes]	level-1
37	Satti, S.A. et. al. 2013	Retrospective study (single city) [one center]	5 yrs. (long)	80 (8 months-14 yrs.) [1.22:1]	Yes (single) [T1DM and DKA]	Unknown	level-3
38	Naeem, M.A., et al. 2015	Retrospective study (single city) [one center]	13 yrs. (long)	373 (8-13 yrs.) [NG]	Yes (single) [T1DM and DKA]	Unknown	level-3
39	Habib, H.S., 2005	Retrospective study (single city) [one center]	12 yrs. (long)	311 (4 mon - 14 yrs.) [152:159]	Yes (single) [T1DM and DKA]	Unknown	level-3
40	Abdul-Rasoul, M., et al. 2010	Retrospective study (nationwide) [multiple center]	6 yrs. (long)	677 (< 12 yrs.) [NG]	Yes (single) [T1DM and DKA]	Unknown	level-3
41	Kulaylat, N.A. et. al. 2001	Retrospective study (single province) [one center]	11 yrs. (long)	46 children (9 yrs.) [27 girls and 19 boys]	Yes (single) [T1DM and DKA]	Unknown	level-3
42	Sayed, M.H., et al. 2017	Retrospective study (single province) [one center]	14 yrs. (long)	NA	yes (unknown) [T1DM and DKA]		level-3
43	Stancakova, A. 2016	Molecular study	NA	NA	yes (unknown) [T2DM]		level-1

44	O'Beirne, S.L., et al. 2016	Molecular study		T2DM - 1124: Normal 590	yes (unknown) [T2DM]	Unknown	level-1
