# Supplementary material for: A Systematic Review of Childhood Diabetes Research in the Middle East Region
Source: Front Endocrinol (Lausanne). 2019 Nov 19;10:805. doi: 10.3389/fendo.2019.00805 (PMC6882272; doi:10.3389/fendo.2019.00805)
Supplement: Supplementary file 5 [file Data_Sheet_5.PDF]

A Systematic Review of Childhood Diabetes Research in the Middle East Region

Saras Saraswathi[1#], Sara Al-Khawaga[1, 3#], Naser Elkum[2] and Khalid Hussain[1*]
[1]Department of Pediatrics, Division of Endocrinology, Sidra Medicine, Doha, Qatar
[2] Clinical Research Center, Biostatistics Sec, Research Services, Sidra Medicine, Doha, Qatar
[3] College of Health & Life Sciences, Hamad Bin Khalifa University, Qatar Foundation, Education City, Doha, Qatar

Appendix E – Quantitative Analysis of Collaborative studies.

This table shows the number of collaborative research studies in the Middle East, with studies involving only one country (28 studies) to some that involve many countries, the highest being 7 countries in one of the studies (Figure 5 in the main manuscript). In total, there are 28 studies involving only one country, 8 studies with two countries in collaboration, 4 studies with 3 collaborating countries each, 2 studies with 4 collaborating countries and one study with 7 collaborating countries.

#	Country	Number of collaborations	Author (year)	Type of study
1	Middle East	7	Habeb et. al. (2012)	Molecular study
2	Qatar	**1**	Alyafei F. et.al. (2018)	Clinical
3	Iran	**1**	Aletayeb MA, et.al. 2018	Clinical
4	Saudi Arabia	**1**	Al-Ghamdi A.H. et. al. 2018	Clinical
5	Saudi Arabia	**1**	Al-Herbish, A.S., et al., 2008	Clinical
6	Kuwait	4	Shaltout, A.A., et al. 2018	Clinical
7	Saudi Arabia	2	Damanhouri, L.H., et al., 2005	Clinical
8	Qatar	2	Alyafei, F., et al. 2018	Clinical
9	Saudi Arabia	2	Al-Jenaidi, F.A., et al. 2005	Clinical
10	Saudi Arabia	**1**	Albishi, M.M.A., 2017	Clinical

11	Saudi Arabia	**1**	Al-Agha, A., A. 2012	Clinical
12	UAE	**1**	Punnose, J., et al. 2002	Clinical
13	UAE	**1**	Punnose, J. et. al. 2005	Clinical
14	Kuwait	**1**	Moussa, M.A., et al. 2008	Clinical
15	Egypt	**1**	Ali, B.A., et al. 2013	Clinical
16	Saudi Arabia	3	Habeb AM et. al. 2012	Molecular study
17	UAE	3	Deeb, A., et al. 2016	Molecular study
18	Iran	2	Abbasi, F., et al. 2018	Molecular study
19	Oman	2	Al Senani, A., et al. 2018	Molecular study
20	Saudi Arabia	3	Elkholy, S. and A.A. Lardhi 2015	Literature Search
21	UAE	**1**	Hussain, T., et al. 2017	Clinical

22	UAE	**1**	Asma, D., et al. 2015	Technology evaluation
23	Saudi Arabia	**1**	Al-Agha, A.E., et al. 2017	Technology evaluation
24	Saudi Arabia	2	Alamoudi, R., et al.2014	Clinical
25	Qatar	**1**	Petrovski, G., et al. 2018	Technology evaluation
26	Qatar	**1**	Petrovski, G., et al. 2018	Technology evaluation
27	Saudi Arabia	4	Cherian, M.P., et al. 2010	Clinical
28	Saudi Arabia	**1**	Abduljabbar, M.A., et al. 2010	Clinical
29	Saudi Arabia	**1**	Habeb, A.M., et al. 2011	Clinical
30	Saudi Arabia	**1**	Al-Rubeaan, K., 2015	Clinical
31	Kuwait	**1**	Moussa, M.A., et al. 2005	Clinical
32	Qatar	2	Alyafei, F., et al. 2018	Clinical

33	Turkey	**1**	Saruhan-Direskeneli, G., et al. 2000	Molecular study
34	Bahrain	3	Al-Harbi, E.M., et al. 2004	Molecular study
35	Saudi Arabia	**1**	Al-Hayek, A.A., et al. 2015	Clinical
36	Kuwait	**1**	Shaltout, A.A., et al. 2016	Clinical
37	Saudi Arabia	**1**	Satti, S.A. et. al. 2013	Clinical
38	Saudi Arabia	**1**	Naeem, M.A., et al. 2015	Clinical
39	Saudi Arabia	**1**	Habib, H.S., 2005	Clinical
40	Kuwait	**1**	Abdul-Rasoul, M., et al. 2010	Clinical
41	Saudi Arabia	**1**	Kulaylat, N.A. et. al. 2001	Clinical
42	Saudi Arabia	**1**	Sayed, M.H., et al. 2017	Clinical
43	Qatar	2	O'Beirne, S.L., et al. 2016	Molecular study
